# TRIM33 plays a critical role in regulating dendritic cell differentiation and homeostasis by modulating *Irf8* and *Bcl2l11* transcription

**DOI:** 10.1038/s41423-024-01179-1

**Published:** 2024-05-31

**Authors:** Xiangyi Shen, Xiaoguang Li, Tao Wu, Tingting Guo, Jiaoyan Lv, Zhimin He, Maocai Luo, Xinyi Zhu, Yujie Tian, Wenlong Lai, Chen Dong, Xiaoyu Hu, Li Wu

**Affiliations:** 1 Institute for Immunology, School of Basic Medical Sciences, Tsinghua University, 100084 Beijing, China; 2 Joint Graduate Program of Peking-Tsinghua-National Institute of Biological Sciences, School of Life Sciences, Tsinghua University, 100084 Beijing, China; 3 Tsinghua-Peking Center for Life Sciences, Tsinghua University, 100084 Beijing, China; 4 Beijing Key Laboratory for Immunological Research on Chronic Diseases, 100084 Beijing, China; 5 Westlake University School of Medicine, Hangzhou, 310024 China

**Keywords:** Dendritic cell, TRIM33, Transcription regulation, IRF8, BIM, Conventional dendritic cells, Plasmacytoid dendritic cells, Myelopoiesis, Immune cell death, Antigen-presenting cells

## Abstract

The development of distinct dendritic cell (DC) subsets, namely, plasmacytoid DCs (pDCs) and conventional DC subsets (cDC1s and cDC2s), is controlled by specific transcription factors. IRF8 is essential for the fate specification of cDC1s. However, how the expression of *Irf8* is regulated is not fully understood. In this study, we identified TRIM33 as a critical regulator of DC differentiation and maintenance. TRIM33 deletion in *Trim33*^fl/fl^ Cre-ER^T2^ mice significantly impaired DC differentiation from hematopoietic progenitors at different developmental stages. TRIM33 deficiency downregulated the expression of multiple genes associated with DC differentiation in these progenitors. TRIM33 promoted the transcription of *Irf8* to facilitate the differentiation of cDC1s by maintaining adequate CDK9 and Ser2 phosphorylated RNA polymerase II (S2 Pol II) levels at *Irf8* gene sites. Moreover, TRIM33 prevented the apoptosis of DCs and progenitors by directly suppressing the PU.1-mediated transcription of *Bcl2l11*, thereby maintaining DC homeostasis. Taken together, our findings identified TRIM33 as a novel and crucial regulator of DC differentiation and maintenance through the modulation of *Irf8* and *Bcl2l11* expression. The finding that TRIM33 functions as a critical regulator of both DC differentiation and survival provides potential benefits for devising DC-based immune interventions and therapies.

## Introduction

Dendritic cells (DCs) are sentinels of the immune system and are crucial regulators of both innate and adaptive immunity. Originating from hematopoietic stem cells (HSCs) and progenitors in the bone marrow (BM), DCs are widely distributed within lymphoid and peripheral nonlymphoid tissues in both mice and humans [1]. DCs can be classified into conventional antigen-presenting DCs (cDCs) and type I interferon (IFN)-producing plasmacytoid DCs (pDCs) [2]. The cDCs contain the cDC1 and cDC2 subsets. cDC1s have an efficient antigen cross-presentation capacity that is crucial for inducing CD8^+^ T-cell responses in antiviral and antitumor immunity [2]. cDC2s are important for inducing helper T-cell responses [2]. Perturbation of DC homeostasis in humans and mice could result in severe immunodeficiencies, including increased susceptibility to infections and neoplasms, and autoimmune conditions [2–5]. Hence, maintaining the homeostasis of DCs is pivotal and involves the regulation of DC differentiation and maintenance.

The development and fate specification of different DC subsets are precisely controlled by multiple transcription factors (TFs) to generate functionally distinct subsets. Some TFs, such as Ikaros and PU.1, are required for the differentiation of all DC subsets [6–9]. Others are crucial for the differentiation of specific DC subsets, e.g., IFN regulatory factor 8 (IRF8), BATF3, ID2, NFIL3, ETV6, and DC-SCRIPT are required for cDC1 differentiation [10–18], while IRF4 and KLF4 are required for cDC2 differentiation [19, 20]. E2-2, ZEB2 and BCL-11A are essential for the differentiation of pDCs, and RUNX2 is required for their exit from the BM [21–25]. Strict regulation of the expression of these TFs is essential for maintaining DC homeostasis. In particular, some recent studies have shown that the expression of *Irf8* in as early as lymphoid-primed multipotent progenitors (LMPPs) potentiates their differentiation toward cDC1s [26, 27]. In terminally differentiated cDC1s, IRF8 also maintains chromatin accessibility, transcriptome, and functionality in the cDC1 signature [28]. The regulation of *Irf8* expression in the DC lineage involves the switching of *cis*-acting superenhancers [29]: the differentiation of cDC1s is dependent on a + 41 kb enhancer of *Irf8* [15], while switching to a + 32 kb enhancer promotes cDC1 maturation [11, 14–16]. However, how these precise regulatory effects are achieved remains to be further explored. The survival of differentiated DCs, on the other hand, is reported to be sustained by the collective expression of members of the BCL-2 family of antiapoptotic proteins, BCL-2, MCL-1 and A1 [30–32], but how the expression of cell death-promoting molecules is suppressed to maintain the survival of DCs requires further investigation.

Tripartite motif-containing 33 (TRIM33), also known as transcription intermediary factor 1-γ (TIF1-γ), is a multifunctional protein. The molecular functions of TRIM33 include roles as a E3 ubiquitin ligase, transcription co-factor, and signaling molecule in the noncanonical TGF-β pathway [33–36]. TRIM33 is a pivotal regulator of the homeostasis and functions of multiple immune cell types [36–40]. In particular, for the myeloid lineage, TRIM33 inhibits the excess expansion of granulocyte-macrophage progenitors (GMPs) and granulocytes [37, 41, 42] and supports proper monocyte and macrophage differentiation and activation [41, 43–45]. However, whether TRIM33 is also involved in the differentiation and function of the DC lineage is still unknown. TRIM33 deficiency leads to a significant reduction in the number of common myeloid progenitors (CMPs) [37], suggesting a potential role for TRIM33 in the development of the DC lineage, which prompted us to investigate the function of TRIM33 in the differentiation of DCs.

In this study, we revealed a novel role of TRIM33 in both the differentiation and maintenance of the DC lineage. Ablation of TRIM33 in DC progenitors significantly impaired their ability to differentiate into DCs, leading to profound defects in the development of cDC1s and pDCs and a relatively moderate reduction in the number of cDC2s. Mechanistic analysis revealed that the deletion of TRIM33 in DC progenitors downregulated the expression of multiple genes associated with DC differentiation, particularly *Irf8*. Furthermore, coimmunoprecipitation (co-IP) and genomic binding site analyses demonstrated that TRIM33 was primarily engaged in binding to active enhancer sites and within transcription complexes at the *Irf8* gene body and at superenhancers and facilitated the transcription of *Irf8* through interactions with cyclin-dependent kinase 9 (CDK9), a key component of positive elongation factor b (P-TEFb). Moreover, TRIM33 colocalized with PU.1 to both facilitate and repress the PU.1-dependent transcriptional regulation of multiple genes, including DC signature genes. More specifically, the deletion of TRIM33 in the DC lineage exaggerated PU.1-dependent *Bcl2l11* enhancer activation and cell apoptosis, which led to a reduction in DC numbers and impaired DC-mediated immune responses. Knockdown of *Bcl2l11* alone in TRIM33-deficient DCs could partially restore DC survival and homeostasis in vivo, while *Irf8* overexpression combined with *Bcl2l11* inactivation significantly restored cDC1 development from TRIM33-deficient BM precursors. Taken together, we identified TRIM33 as a new critical regulator of DC development and maintenance by modulating the transcription of *Irf8* and the expression of *Bcl2l11*.

## Results

### TRIM33 was required for maintaining DC homeostasis

**Fig. 1:**
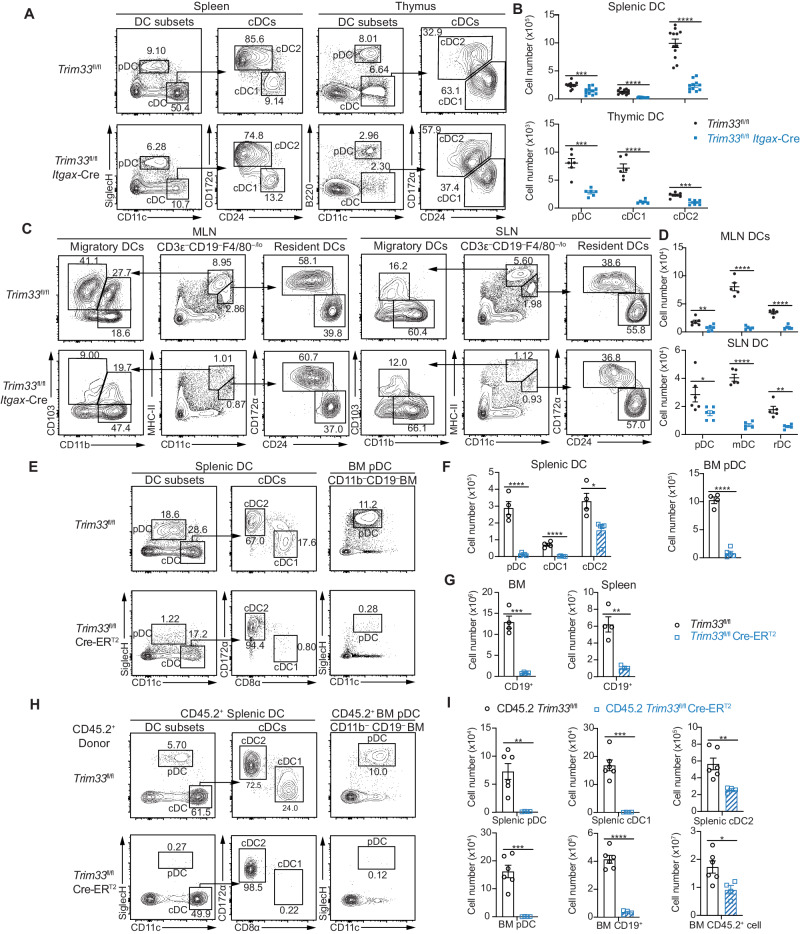
TRIM33 deficiency resulted in cell-intrinsic impairments of DC homeostasis and development. **A–D** Flow cytometry analysis of DC populations in *Trim33*^fl/fl^ and *Trim33*^fl/fl^ *Itgax*-Cre mice. The samples in (A) were enriched for DCs, and viable cells were pregated. MLN: mesenteric lymph node, SLN: subcutaneous lymph node. **B**, **D** Numbers of splenic CD11c^int^SiglecH^+^ pDCs, CD11c^+^SiglecH^–^CD24^+^CD172α^–^ cDC1s, and CD11c^+^SiglecH^–^CD24^–^CD172α^+^ cDC2s; thymic CD11c^int^B220^+^ pDCs, CD11c^+^B220^–^CD24^+^CD172α^–^ cDC1s, and CD11c^+^B220^–^CD24^–^CD172α^+^ cDC2s; and lymph node B220^+^PDCA-1^+^ pDCs (Fig. S2A), CD3ε^–^CD19^–^F4/80^–/lo^CD11c^+^MHC-II^hi^ migratory DCs (mDCs), and CD3ε^–^CD19^–^F4/80^–/lo^CD11c^hi^MHC-II^+^ resident DCs (rDCs). **E-G** Flow cytometry analysis of splenic DCs and BM pDCs from tamoxifen-treated *Trim33*^fl/fl^ and *Trim33*^fl/fl^ Cre-ER^T2^ mice. DC-enriched splenocytes were pregated for viable cells, and BM cells were pregated for viable CD11b^–^CD19^–^ cells. Plots in (**F**) show the numbers of splenic pDCs, SiglecH^–^CD11c^+^CD8α^+^CD172α^–^ cDC1s, SiglecH^–^CD11c^+^CD8α^–^CD172α^+^ cDC2s, and SiglecH^+^CD11c^+^ BM pDCs. **G** Numbers of BM and splenic CD19^+^ cells. *N* = 4–5. **H**, **I** Flow cytometry analysis of CD45.2^+^ populations in the indicated BM-reconstituted mice. Mice were treated with tamoxifen 3 weeks after reconstitution. Viable CD45.2^+^ cells were gated as described in (**E**). *N* = 4–6. The numbers on the plots indicate percentages. The absolute cell number per animal is shown. Bars represent the means ± SEMs. Representative data from 2 to 3 independent experiments are shown. The data were analyzed using two-tailed Student’s *t* tests. *P < 0.05, **P < 0.01, ***P < 0.001, and ****P < 0.0001

TRIM33 is expressed by many immune cell types, including DCs and DC progenitors (Fig. S1A, B). We first crossed *Trim33*^fl/fl^ (wild-type, WT) [36, 46] mice with *Itgax-*Cre [47] mice to conditionally delete *Trim33* in differentiated DCs of *Trim33*^fl/fl^ *Itgax-*Cre mice (conditional knockout, cKO) and evaluate the role of TRIM33 in DCs (Fig. S1C). *Trim33* was efficiently and specifically inactivated in CD11c-expressing peripheral pDCs and cDCs of cKO mice but not in BM pre-DCs or pDCs (Fig. S1D–G), resulting in a significant disturbance of DC homeostasis in lymphoid tissues. While the numbers of total splenocytes and non-CD11c-expressing lymphocytes in cKO mice were unaffected (Fig. S1H–J), the numbers of all pDCs and cDC populations were significantly reduced in the spleen, thymus, and lymph nodes of cKO mice (Fig. 1A–D, Fig. S2A, B). BM chimeric mice were generated by coinjecting CD45.2 WT or cKO BM together with CD45.1 wild-type BM cells for DC generation in vivo to further determine whether TRIM33 plays a cell-intrinsic role in DCs. The numbers of all three DC subsets derived from CD45.2 cKO BM were significantly reduced, whereas no significant changes in the numbers of other lineages of cells derived from cKO BM were found (Fig. S2C, D). These results indicated that TRIM33 was required for maintaining DC homeostasis.

As a consequence of impaired DC homeostasis, DC-dependent immune responses were also hindered in cKO mice (Fig. S3). The cKO mice displayed deficiencies in antigen-specific T-cell priming (Fig. S3A–C) and rapid IFN-α production, IFN-γ generation, and viral clearance following acute lymphocytic choriomeningitis virus (LCMV) infection in vivo (Fig. S3D–F).

### TRIM33 was required for cell-intrinsic DC development

We generated *Trim33*^fl/fl^ Cre-ER^T2^ mice, in which *Trim33* can be systemically deleted after tamoxifen treatment, to further investigate whether TRIM33 is also required in earlier stages of DC development (Fig. S4A, B). Both in vivo tamoxifen administration and in vitro treatment of BM cultures with 4-hydroxytamoxifen (4-OHT) elicited efficient *Trim33* deletion (Fig. S4C, D). Remarkably, tamoxifen treatment led to an almost complete loss of cDC1s and pDCs and a moderate reduction in the number of cDC2s in the spleen and BM of *Trim33*^fl/fl^ Cre-ER^T2^ mice (Fig. 1E, F). The lack of cDC1s and pDCs in *Trim33*^fl/fl^ Cre-ER^T2^ mice was not due to the altered expression of the used DC markers, since additional staining with XCR1 and CD24 for cDC1s and with CD45RA, PDCA-1 and B220 for pDCs was also performed to confirm the phenotype (Fig. S4E–G). The more drastic reduction in DCs in *Trim33*^fl/fl^ Cre-ER^T2^ mice than in *Trim33*^fl/fl^ *Itgax-*Cre mice suggested that TRIM33 might also play a crucial role in the development of DCs from an early stage. Consistent with previous reports [37, 40–43, 48], we also observed decreased numbers of B cells, monocytes, and macrophages and increased numbers of granulocytes in *Trim33*^fl/fl^ Cre-ER^T2^ mice (Fig. 1G, Fig. S4H). A moderate decrease in the number of NK cells was also detected (Fig. S4H).

We generated BM chimeric mice by adoptively transferring BM cells from CD45.2 *Trim33*^fl/fl^ or CD45.2 *Trim33*^fl/fl^ Cre-ER^T2^ mice together with CD45.1 wild-type BM cells to lethally irradiated CD45.1 recipients to determine which cellular compartment, i.e., hematopoietic or nonhematopoietic, contributed to the loss of cDC1s and pDCs in *Trim33*^fl/fl^ Cre-ER^T2^ mice after tamoxifen-induced TRIM33 deletion. Three weeks after transplantation, the chimeric mice were treated with tamoxifen for 5 consecutive days and analyzed after 7 additional days for CD45.2 donor BM-derived DC subsets (Fig. S4B). CD45.2 *Trim33*^fl/fl^ BM cells were able to reconstitute pDCs in the BM and all 3 subsets of DCs in the spleen of recipient mice (Fig. 1H, I). In contrast, CD45.2 *Trim33*^fl/fl^ Cre-ER^T2^ BM generated barely detectable cDC1 and pDC subsets and a reduced number of cDC2s, which recapitulated the DC defects of nonchimeric *Trim33*^fl/fl^ Cre-ER^T2^ mice (Fig. 1H, I). Moreover, the CD45.2 *Trim33*^fl/fl^ Cre-ER^T2^ BM cells showed defective competition after tamoxifen treatment (Fig. 1I), which was consistent with the reported defects in BM self-renewal and the retention of TRIM33-deficient HSCs and progenitors [37]. In contrast, the reconstitution of DC populations and other immune cells by CD45.1 WT donor BM was comparable in chimeric mice with either tamoxifen-treated CD45.2 *Trim33*^fl/fl^ or CD45.2 *Trim33*^fl/fl^ Cre-ER^T2^ mice as recipients (Fig. S5), further indicating a cell autonomous role for TRIM33. Collectively, these results confirmed the hematopoietic cell-intrinsic roles of TRIM33 in DC development and homeostasis.

Single-cell RNA sequencing (scRNA-seq) analysis identified novel human DC subsets, including cDC2a, cDC2b, and AXL^+^ DCs [49]. We further examined whether TRIM33 plays a role in the development of the murine counterparts of these DC subsets. We found that the proportions of the cDC2, cDC2a and cDC2b subsets [50] were also decreased by CD11c-conditoned *Trim33* deletion (Fig. S6A, B). The numbers of noncanonical DCs and AXL^+^ transitional DCs (tDCs) [51] were unchanged in *Trim33*^fl/fl^ *Itgax*-Cre mice (Fig. S6C, D) but were significantly reduced in tamoxifen-treated *Trim33*^fl/fl^ Cre-ER^T2^ mice (Fig. S6E, F), suggesting a dependence on TRIM33 for the development of noncanonical DCs and tDCs from their early precursors or incomplete TRIM33 deletion in the former model, as CD11c levels in these cells were relatively low [51]. These results further confirmed that TRIM33 was required for the homeostasis of all DCs.

An Flt3L (FL, FMS-like tyrosine kinase 3 ligand) in vitro culture system for DC generation [52] was utilized with the addition of 4-OHT to determine the kinetics of DC generation from the BM cells of *Trim33*^fl/fl^ Cre-ER^T2^ mice and further determine the role of TRIM33 in DC development. As shown in Fig. S7, *Trim33*^fl/fl^ BM readily generated increasing numbers of cDC1s, cDC2s, and pDCs with time, while *Trim33*^fl/fl^ Cre-ER^T2^ BM generated significantly fewer numbers of all three DC subsets at all the timepoints detected. Taken together, these results indicated that TRIM33 deficiency resulted in a cell-intrinsic impairment of DC development.

### TRIM33 was required for maintaining the homeostasis and DC differentiation potential of early hematopoietic progenitors

**Fig. 2:**
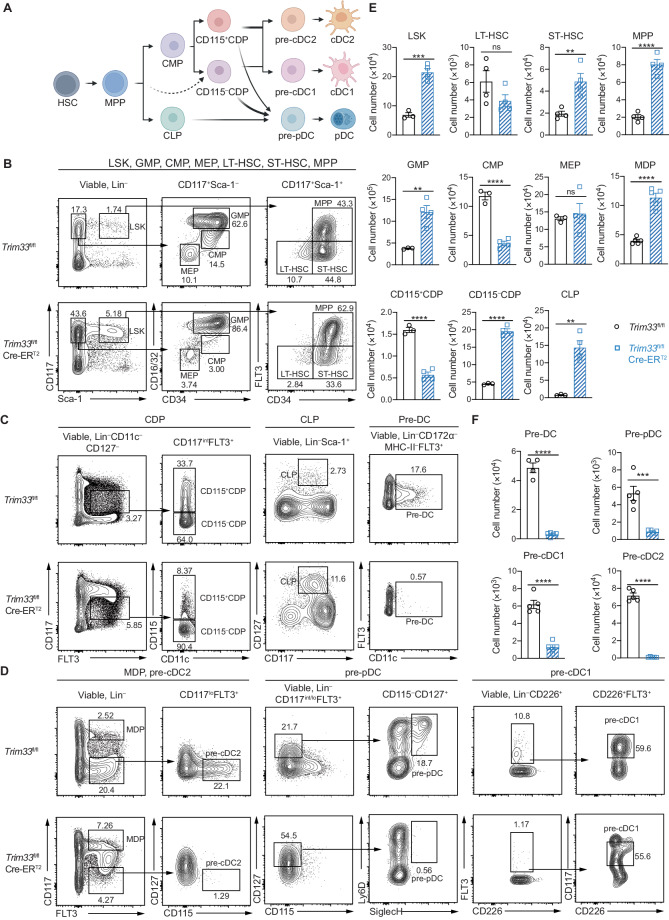
TRIM33 deficiency disrupted the homeostasis of DC progenitors in the bone marrow. **A** Schematic plot indicating the progenitors and pathways involved in the development of different DC subsets. HSC hematopoietic stem cell, MPP multipotent progenitor, CMP common myeloid progenitor, CLP common lymphoid progenitor, CDP common DC progenitor. The solid lines show confirmed developmental pathways, and the dashed line indicates a developmental relationship yet to be confirmed. The figure was created with biorender.com. **B–D** Flow cytometry analysis of BM progenitors. BM cells from *Trim33*^fl/fl^ or *Trim33*^fl/fl^ Cre-ER^T2^ mice were pregated for viability and negative staining for lineage markers (Lin^–^ i.e., CD2^–^CD3^–^CD8^–^CD11b^–^CD19^–^B220^–^TER119^–^Ly6G^–^). Different progenitors were then defined by various surface markers: LSKs—Lin^–^Sca-1^+^CD117^hi^, long-term HSCs (LT-HSCs)—Lin^–^Sca-1^+^CD117^hi^CD34^–^FLT3^–^, short-term HSCs (ST-HSCs)—Lin^–^Sca-1^+^CD117^hi^CD34^+^FLT3^–^, MPPs—Lin^–^Sca-1^+^CD117^hi^CD34^+^FLT3^+^, GMPs—Lin^–^Sca-1^–^CD117^hi^CD34^+^CD16/32^hi^, CMPs—Lin^–^Sca-1^–^CD117^hi^CD34^+^CD16/32^lo^, MEPs (megakaryocyte-erythroid progenitors)—Lin^–^Sca-1^–^CD117^hi^CD34^–^CD16/32^–^, CD115^+^ CDPs—Lin^–^CD11c^–^CD127^–^CD117^int^FLT3^+^CD115^+^, CD115^–^ CDPs—Lin^–^CD11c^–^CD127^–^CD117^int^FLT3^+^CD115^–^, CLPs—Lin^–^Sca-1^+^CD117^+^CD127^+^, MDPs—Lin^–^CD117^hi^FLT3^+^, pre-DCs—Lin^–^CD172α^–^MHC-II^–^FLT3^+^CD11c^+^, pre-pDCs—Lin^– ^CD117^int/lo^FLT3^+^CD115^–^CD127^+^Ly6D^+^SiglecH^+^, pre-cDC1s—Lin^–^CD226^+^FLT3^+^CD117^int^, and pre-cDC2s—Lin^–^CD117^lo^FLT3^+^CD115^+^. **E**, **F** The numbers (means ± SEMs) of the indicated progenitors per animal. *N* = 3–5. Representative data from 2-3 independent experiments are shown. The data were analyzed using two-tailed Student’s *t* tests. Ns: *P* > 0.05, **P < 0.01, ***P < 0.001, and ****P < 0.0001

Since BM cells contain different progenitors with varying DC differentiation potential, we then examined the effects of *Trim33* deletion on these progenitors in *Trim33*^fl/fl^ Cre-ER^T2^ mice treated with tamoxifen. As shown in Fig. 2A, multipotent progenitors (MPPs), the downstream progenitors of HSCs, generate common lymphoid progenitors (CLPs) and CMPs. The latter then give rise to common DC progenitors (CDPs) containing CD115^+^ and CD115^–^ subsets [53]. cDCs can be generated predominantly from CD115^+^CDPs via pre-cDCs, whereas pDCs can be produced by both CLPs and CD115^–^CDPs [54–57]. We found that the proportions and numbers of these progenitors were altered significantly in *Trim33*^fl/fl^ Cre-ER^T2^ mice after tamoxifen treatment, indicating a severe distortion and developmental blockade of DC differentiation (Fig. 2B–F). Compared to *Trim33*^fl/fl^ mice, tamoxifen-treated *Trim33*^fl/fl^ Cre-ER^T2^ mice exhibited significant increases in the numbers of short-term HSCs (ST-HSCs), MPPs, GMPs, presumed CD117^hi^FLT3 ^+^ MDPs (macrophage–DC progenitors), CLPs and CD115^–^ CDPs and marked decreases in the numbers of CMPs, CD115^+^ CDPs, CD117^int/lo^FLT3^+^CD115^–^CD127^+^Ly6D^+^SiglecH^+^ prepDCs, CD117^int^CD226^+^FLT3^+^ pre-cDC1s, CD117^lo^FLT3^+^CD115^+^ pre-cDC2s, and Lin^–^CD172α^–^FLT3^+^CD11c^+^MHC-II^–^ pre-DCs [11, 58, 59] (Fig. 2B–F). The expansion of ST-HSCs, MPPs and GMPs and the reduction in CMPs in tamoxifen-treated *Trim33*^fl/fl^ Cre-ER^T2^ mice were in accordance with the results reported in a previous study [37].

**Fig. 3:**
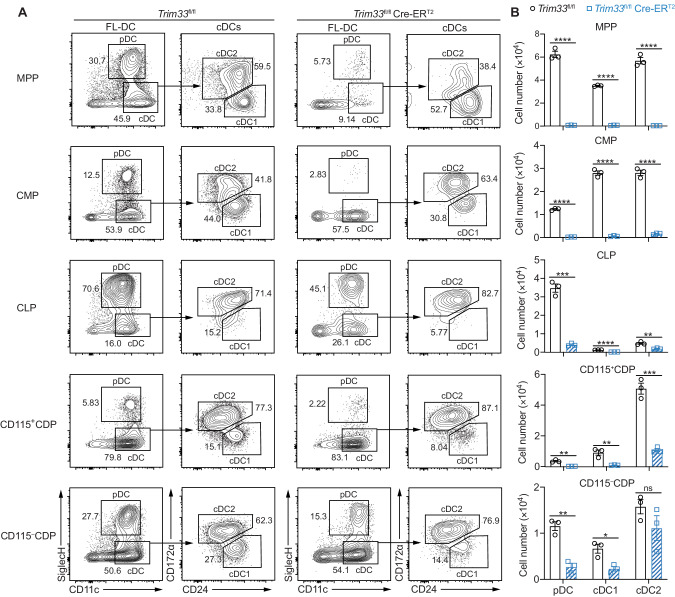
TRIM33-deficient progenitors exhibited an impaired DC generation capacity in vitro. **A** Flow cytometry analysis of DCs generated from different progenitors in Flt3L cultures. CD45.2^+^ MPPs (Lin^–^CD117^+^Sca-1^+^CD34^+^FLT3^+^), CMPs (Lin^–^CD117^+^Sca-1^–^CD16/32^lo^CD34^+^), CLPs (Lin^–^CD11c^–^CD117^int^FLT3^+^CD127^+^), CD115^+^ CDPs (Lin^–^CD11c^–^CD117^int^FLT3^+^CD127^–^CD115^+^), and CD115^–^ CDPs (Lin^–^CD11c^–^CD117^int^ FLT3^+^CD127^–^CD115^–^) were sorted by FACS from the BM of mice of the indicated genotypes and cultured with CD45.1^+^ BM feeder cells in medium supplemented with 200 ng/mL Flt3L and 1 µM 4-OHT. Cultures with MPPs, CMPs and CLPs, and CDPs were analyzed on Day 9, Day 5, and Day 4, respectively. Plots were pregated on viable and CD45.2^+^ cells. Numbers adjacent to gates indicate the percentages of the parent population. **B** Numbers of DCs generated per 1$\times$10^4^ input progenitors are shown. *N* = 3. The data are representative of 2–3 independent experiments. Bars represent the means ± SEMs. The data were analyzed using two-tailed Student’s t tests. Ns not significant, *P < 0.05, **P < 0.01, ***P < 0.001, and ****P < 0.0001

Although the numbers of CMPs and CD115^+^ CDPs were decreased, which might partly account for the loss of the cDC and pDC subsets, the increased numbers of MPPs, CLPs and CD115^–^ CDPs seemed contradictory to the reduced numbers of DCs in TRIM33-deficient mice. We determined the DC generation capacity of each purified progenitor population (Fig. S8) in vitro by Flt3L culture to address this issue (Fig. 3). The capacities of TRIM33-deficient MPPs, CMPs and CD115^+^ CDPs to generate both cDCs and pDCs and the ability of TRIM33-deficient CLPs to generate pDCs were substantially impaired (Fig. 3A, B). TRIM33-deficient CD115^–^ CDPs exhibited an impaired ability to produce pDCs and cDC1s but generated a comparable number of cDC2s to WT CD115^–^ CDPs (Fig. 3A, B). These results showed that despite the increased numbers, the DC generation capacities of TRIM33-deficient MPPs, CLPs and CD115^–^ CDPs were substantially impaired. Taken together, our findings indicated that TRIM33 was required for the development of DCs from progenitors at different developmental stages.

Noticeably, TRIM33-deficient MPPs and CMPs exhibited severe defects in generating all DC progenies in Flt3L cultures (Fig. 3A, B), which seemed discordant with the in vivo phenotype of a moderate reduction in cDC2s. This finding suggested that TRIM33 deficiency might affect the maintenance of DC progenitors in culture. By culturing BM progenitors purified from *Trim33*^fl/fl^ and *Trim33*^fl/fl^ Cre-ER^T2^ mice in vitro with or without supporting growth factors, including stem cell factor (SCF) and Flt3L, we found that TRIM33 deficiency resulted in survival and expansion defects in all DC progenitors, including LSKs (Lin^–^Sca-1^+^CD117^+^, containing HSCs and MPPs), CMPs, CLPs, CD115^+^ CDPs, CD115^–^ CDPs and pre-DCs, but not in non-DC-producing GMPs (Fig. S9). Among the DC progenitors, the survival of the TRIM33-deficient CLPs was most strongly impaired (Fig. S9). Taken together, TRIM33 is indispensable for the maintenance of DC progenitors.

### TRIM33 was required for the expression of multiple DC-associated genes in DC progenitors

**Fig. 4:**
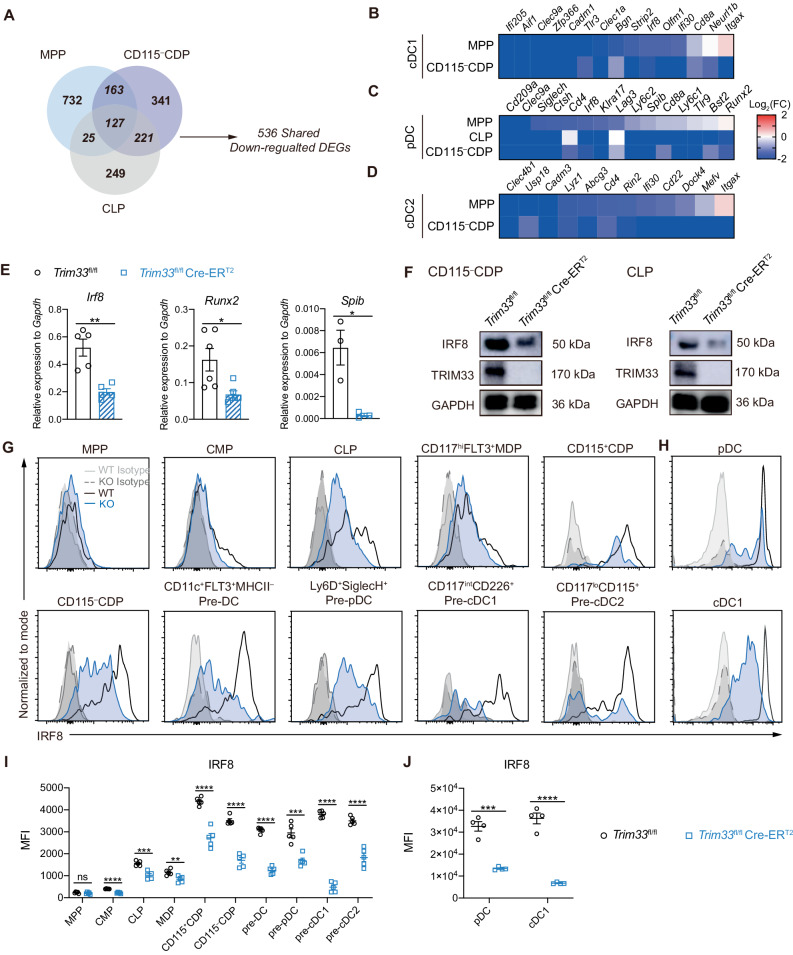
TRIM33 deficiency altered the expression of multiple DC-associated genes in progenitors. **A** Venn diagram indicating the RNA-seq-detected differentially expressed genes (DEGs) downregulated by *Trim33* deletion in the MPPs, CD115^–^ CDPs and CLPs. A DEG was considered shared if it was downregulated in more than one population. Heatmap presenting the altered expression of (**B**) cDC1-associated, (**C**) pDC-associated, and (**D**) cDC2-associated genes in progenitors of tamoxifen-treated *Trim33*^fl/fl^ Cre-ER^T2^ (KO) mice compared to their *Trim33*^fl/fl^ (WT) counterparts. Log_2_(KO to WT fold change) values of the FPKM values are plotted. **E** qPCR analysis detected the mRNA levels of *Irf8*, *Runx2* and *Spib* in CD115^–^ CDPs from the indicated genotypes. *N* = 3–6. **F** Western blot analysis of IRF8 expression in CD115^–^ CDPs and CLPs of the indicated genotypes. Flow cytometry analysis of intracellular IRF8 protein levels in the indicated progenitors (**G**) from tamoxifen-treated *Trim33*^fl/fl^ and *Trim33*^fl/fl^ Cre-ER^T2^ mice and from Flt3L culture-generated pDCs and cDC1s of the indicated genotypes (**H**). **I, J** Mean fluorescent intensity (MFI) of IRF8 in the indicated cells; *n* = 4. The data are representative of 2–3 independent experiments. Bars represent the means ± SEMs. The data were analyzed using two-tailed Student’s *t* tests. *P < 0.05, **P < 0.01, ***P < 0.001, and ****P < 0.0001

We performed RNA-seq analysis to reveal the transcriptional profiles of WT and TRIM33-deficient MPPs, CLPs, and CD115^–^ CDPs and explore how TRIM33 deficiency affects the DC differentiation potential of progenitors. Most of the differentially expressed genes (DEGs) identified between WT and TRIM33-deficient progenitors were strongly downregulated by TRIM33 deficiency (Fig. S10A). Among the downregulated DEGs, 536 genes were shared by more than one progenitor populations (Fig. 4A, Table S1) and were closely related to hematopoiesis and immune responses (Fig. S10B, C). More importantly, while the upregulated DEGs showed no apparent association with DC development (Fig. S10D), the 536 shared downregulated DEGs included multiple DC “signature genes” defined in previous studies [9, 12, 58, 60]. These DEGs involved cDC1-associated genes, including *Irf8*, *Zfp366*[DC-SCRIPT], *Itgax*, *Cd8a*, *Ifi205*, and *Tlr3* (Fig. 4B); pDC-associated genes, including *Irf8*, *Runx2, Spib*, *Ly6c1*, *Ly6c2*, *Bst2*, *SiglecH*, and *Cd209a* (Fig. 4C); and cDC2-associated genes, including *Cd22*, *Ifi30*, *Clec4b1*, *Lyz1*, and *Cadm3* (Fig. 4D). However, no alteration in the expression of PU.1, the key transcription factor for DC development, was observed in the progenitors [37] (Fig. S10E). The reduced mRNA levels of *Irf8, Runx2* and *Spib* in TRIM33-deficient CD115^–^ CDPs were then verified by qPCR analysis (Fig. 4E). Thus, TRIM33 is required for the DC differentiation potential of DC progenitors by promoting the expression of multiple DC-associated genes.

IRF8 is an essential TF for DC differentiation, especially for cDC1 lineage fate specification [11, 15]. We found 30 genes (Fig. S10F) among the 536 downregulated DEGs that were specifically induced in DCs by IRF8 [12], suggesting that *Irf8* may account for some of the transcriptional distortions in TRIM33-deficient DC progenitors. As none of the downregulated cDC2-associated genes were essential TFs for cDC2 fate determination and neither RUNX2 nor Spi-B [24, 25, 61] played a role as cardinal as IRF8 for cDC1 differentiation, we focused on whether TRIM33 promoted *Irf8* expression. Consistent with the reduction in mRNA levels, Western blot analysis also revealed a significant reduction in IRF8 at the protein level in TRIM33-deficient CD115^–^ CDPs and CLPs (Fig. 4F). Except for the low *Irf8*-expressing MPPs, intracellular staining further confirmed decreased IRF8 protein levels across all TRIM33-deficient progenitors and precursors, including MDPs, CMPs, CD115^+^ CDPs, CD115^–^ CDPs, CLPs, Ly6D^+^SiglecH^+^ pre-pDCs, CD117^int^CD226^+^FLT3^+^ pre-cDC1s, CD117^lo^FLT3^+^CD115^+^ pre-cDC2s, and FLT3^+^CD11c^+^MHC-II^–^ pre-DCs (Fig. 4G, I). TRIM33-deficient pDCs and cDC1s generated in Flt3L cultures also exhibited downregulated IRF8 protein expression (Fig. 4H, J). These data therefore confirmed that TRIM33 is required for *Irf8* expression in multiple DC progenitors.

### TRIM33 modulated PU.1 transcription function by colocalization at active enhancers

**Fig. 5:**
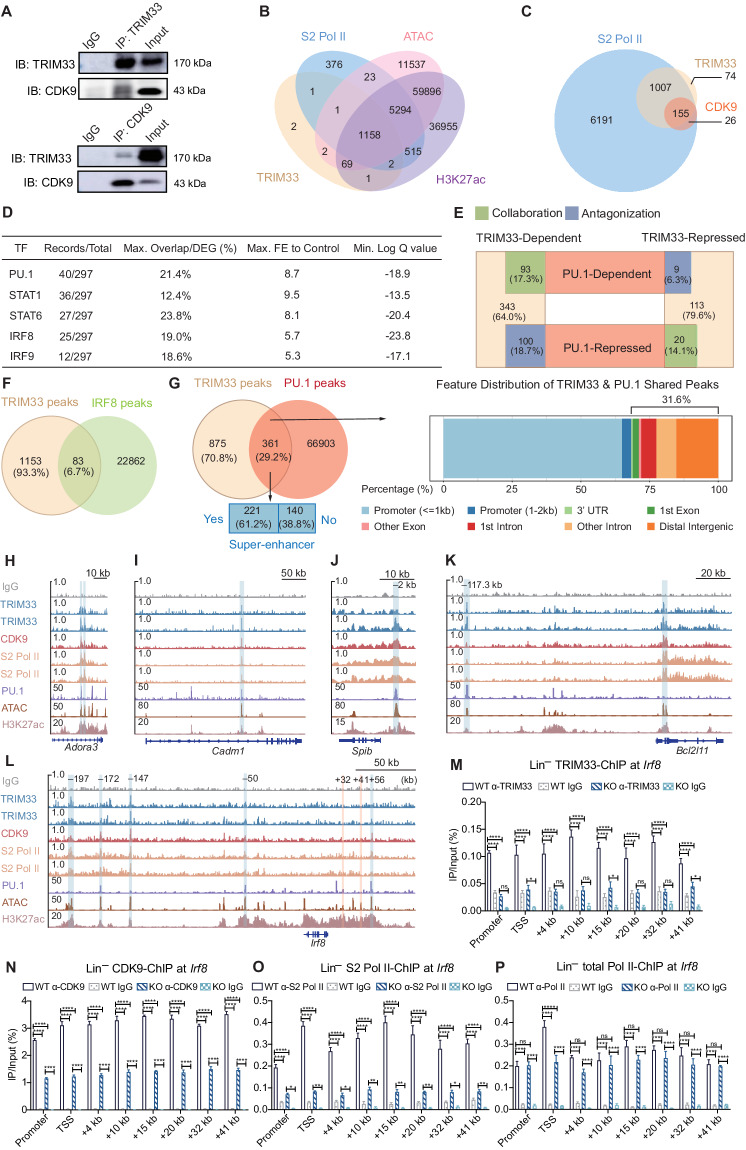
TRIM33 modulated the PU.1 transcriptional function and *Irf8* expression via chromatin occupancy. **A** The interaction of TRIM33 with CDK9 in Mutu DC1940 cells was detected using coimmunoprecipitation. **B**, **C** Venn diagrams indicating the intersection between genomic regions bound by the indicated factors in CDPs. CDP TRIM33, CDK9, and S2 Pol II peaks were called from CUT&Tag data. CDP ATAC-seq and H3K27ac-ChIP region data were retrieved from the GSE132240 and GSE132239 datasets. **D** ChIP-Atlas analysis of RNA-seq-detected progenitor DEGs following TRIM33 deletion. FE, fold enrichment. **E** Intersection between TRIM33-reliant and PU.1-reliant transcriptomes. The percentages of TRIM33-dependent/repressed genes under the control of PU.1 are shown. **F** Intersection between CDP TRIM33 CUT&Tag peaks and cDC1 IRF8-ChIP (GSE66899) peaks. **G** Analysis of shared TRIM33 and PU.1 peaks in CDPs. PU.1-ChIP peaks were called from GSE57563. The superenhancer analysis was performed with SEA v3.0. **H–L** Visualization of TRIM33, CDK9, S2 Pol II CUT&Tag, PU.1-ChIP, H3K27ac ChIP, and ATAC-seq data at the indicated loci in CDPs. The reported +32 kb and +41 kb enhancer sites of *Irf8* are shaded in orange. ChIP‒qPCR analysis of TRIM33 (**M**), CDK9 (**N**), S2 Pol II (**O**), and total Pol II (**P**) occupancy at different regions of the *Irf8* gene in BM Lin^–^ cells from tamoxifen-treated *Trim33*^fl/fl^ (WT) and *Trim33*^fl/fl^ Cre-ER^T2^ (KO) mice. *N* = 3–4. The data are representative of 2 independent experiments. Bars represent the means ± SEMs. The data were analyzed using two-way ANOVA followed by Bonferroni’s multiple comparison test. *P < 0.05, **P < 0.01, ***P < 0.001, and ****P < 0.0001

Mass spectrometry analyses were performed on BM lineage marker-negative (Lin^–^) FLT3^+^ DC precursors to elucidate the potential molecular mechanism of TRIM33 transcriptional regulation. The results revealed that TRIM33-interacting proteins are primarily involved in chromatin assembly, ribosome biogenesis, and RNA metabolism pathways (Table S2, Fig. S11), suggesting that TRIM33 plays a role in chromatin conformational changes and RNA processing. In particular, an interaction between TRIM33 and CDK9, a core subunit of P-TEFb [62], was identified (Fig. S11C). CDK9 phosphorylates the serine 2 residue of the RNA polymerase II (Pol II) C-terminal domain (CTD) for active Pol II transcription [62]. The physical association of TRIM33 and CDK9, verified in both anti-TRIM33- and anti-CDK9-immunoprecipitated lysates of Mutu DC1940 cells [63] (Fig. 5A), may influence S2 Pol II-mediated gene transcription. However, the expression of both CDK9 and Pol II in BM precursors did not seem to be regulated by TRIM33 (Fig. S12A, B).

We therefore investigated the genome-wide binding sites of TRIM33, CDK9, and S2 Pol II in wild-type CDPs via a CUT&Tag assay. As shown in Fig. S12C–F, similar to those of the CDK9 and S2 Pol II signals, the TRIM33 signals were centered on transcription start sites (TSSs) and located primarily in promoter regions, suggesting a transcriptional regulatory role. Remarkably, compared with the H3K27 acetylation (H3K27ac) chromatin immunoprecipitation sequencing (ChIP-seq) and assay for transposase-accessible chromatin sequencing (ATAC-seq) datasets of CDPs [11], 99.5% (1230/1236) of TRIM33 binding sites were located in active enhancer regions denoted by H3K27ac [64], and 93.7% (1158/1236) of TRIM33 regions were colocalized with S2 Pol II on transposase-accessible H3K27ac-enriched chromatin (Fig. 5B). Further analysis revealed that the recruitment of detectable CDK9 to chromatin in the CDPs was substantially reliant on the presence of TRIM33 (Fig. 5C), although only a portion of the S2 Pol II peak intersected with TRIM33. A motif analysis of the TRIM33 peaks revealed possible colocalization with PU.1, in accordance with previous reports [37, 40], and several other TFs with unknown functions in DC development (Fig. S12G).

As the data strongly suggested the active engagement of TRIM33 in the gene transcription of CDPs, especially at enhancer sites, only 1.8% of the genes annotated from the TRIM33 peaks matched the RNA-seq-detected DEGs in progenitors. Suspecting that some of the transcriptome alterations in TRIM33-deficient progenitors were secondary to changes in the expression or function of master regulators, we performed a TSS TF enrichment analysis of the DEGs and found that PU.1 and IRF8 were among the top predicted genes (Fig. 5D). We first hypothesized that the loss of TRIM33 colocalization on chromatin might disturb the transcriptional regulatory function of PU.1. We intersected the DEGs with the PU.1-dependent genes in DCs defined by Chopin et al. [9] to elucidate this possibility and found that 32.7% (222/678) of the TRIM33-regulated genes were under dual control of TRIM33 and PU.1, half by collaboration (50.9%, 113/222) and the other half by antagonization (49.1%, 109/222) (Fig. 5E, Table S3). Importantly, the collaboration of TRIM33 and PU.1 promoted the expression of more cDC-associated genes, while the antagonistic effect of TRIM33 on PU.1-mediated suppression was attributed to the expression of more pDC-associated genes (Fig. S12H). Hence, TRIM33 regulated DC development by influencing the transcriptional regulatory function of PU.1 in DC progenitors.

By intersecting genomic regions occupied by TRIM33 in CDPs to those of PU.1 in CDPs or IRF8 in cDC1s[14, 65], we found that while TRIM33 and IRF8 shared only a few co-occupancy sites (Figs. 5F), 29.2% (361/1236) of TRIM33 peaks were also bound by PU.1 (Fig. 5G, Table S4). Noting that almost all TRIM33 peaks were H3K27ac-enriched sites that are active enhancers, we further revealed with SEA [66] that 61.2% of these shared peaks were superenhancers in mouse CDPs, and 31.6% were located in nonpromoter regions (Fig. 5G). We again noticed that only a few RNA-seq-detected DEGs, such as *Adora3* and *Cadm1*, were consistent with the annotation of the shared peaks of PU.1 and TRIM33 (Fig. S12I, Fig. 5H, I). A visual inspection revealed omissions during data processing, such as an initially unidentified –2 kb peak of *Spib* (Fig. 5J); however, annotating bias of distant enhancer peaks to genes of the greatest proximity instead of regulated genes may be present. EnhancerAtlas 2.0, a tool capable of enhancer–target gene prediction [67], further revealed a potential enhancer of the pro-apoptotic BIM-encoding gene *Bcl2l11* that was 117.3 kb upstream (Fig. 5K). TRIM33 was reported to repress *Bcl2l11* expression in murine B-ALL by antagonizing PU.1 binding at the -117.3 kb enhancer [40]. Considering the accelerated death of TRIM33-deficient progenitors in vitro (Fig. S9) and the elevated *Bcl2l11* transcript level in these cells (Fig. S12J), TRIM33 antagonization of PU.1-directed *Bcl2l11* transcription might be required for DC progenitor survival. Therefore, TRIM33 supports the differentiation and survival of the DC lineage by modulating the transcriptional control of PU.1-regulated genes, including *Bcl2l11*.

### TRIM33 promoted *Irf8* transcription by influencing S2 Pol II chromatin occupancy

We further analyzed the CUT&Tag data by visualizing TRIM33 binding to the *Irf8* locus to understand how a marked decrease in IRF8 levels in TRIM33-deficient progenitors was achieved. Interestingly, although the results revealed little or no significant binding of TRIM33 to the *Irf8* gene body or the known –50 kb, +32 kb, +41 kb, and +56 kb enhancers [11, 68, 69], TRIM33 binding to distal enhancer-like regions, such as –147 kb and possibly –172 kb and –197 kb, was detected (Fig. 5L). Moreover, Capture C data from CDPs [70] indicated that the *cis*-regulatory element of *Irf8* could extend beyond this distance (Fig. S12K). However, whether these sites could be genuine enhancers of *Irf8* that control its expression and in vivo DC development remains to be further investigated. Since CUT&Tag technology has not been validated for co-factor analysis and might underestimate authentic TRIM33 occupancy, we performed TRIM33 ChIP‒qPCR directly targeting the *Irf8* promoter, gene body (TSS to 20 kb), and known +32 kb and +41 kb DC-relevant enhancers in BM Lin^–^ precursors. Relatively weak yet significant TRIM33 occupancy was confirmed at these sites in wild-type cells (Fig. 5M). More importantly, we observed a significant decrease in the occupancy of CDK9, S2 Pol II, and S5 Pol II, but not total Pol II, at these sites following tamoxifen-induced TRIM33 deletion in Lin^–^ precursors (Fig. 5N–P, Fig. S12L). The loss of S2 Pol II, the active transcribing form of Pol II, around the *Irf8* locus due to the absence of TRIM33 and the reduction in CDK9 recruitment significantly reduced the generation of *Irf8* transcripts. Hence, these results support a role for TRIM33 in promoting *Irf8* transcription in DC progenitors and precursors via chromatin occupancy.

Notably, overexpression of *Irf8* alone in TRIM33-deficient BM Lin^–^ cells (Fig. S13A) did not restore cDC1 generation from these cells in Flt3L cultures (Fig. S13B, C). This finding further suggested additional targets of TRIM33 that cooperate with IRF8 in the regulation of DC differentiation and homeostasis.

### TRIM33 maintained DC survival by suppressing *Bcl2l11* transcription

We previously showed a role for TRIM33 in the maintenance of DC progenitor survival and suggested that the repression of *Bcl2l11* by TRIM33 is the underlying mechanism, yet the IRF8 insufficiency-induced developmental impairment, for instance, MDP to CDP and pre-cDC1 to cDC1 transitions [10], might directly elicit cell death. We then explored whether TRIM33 utilizes any IRF8-independent mechanism to promote DC lineage maintenance. Changes in the IRF8 level and cell viability of sorted *Trim33*^fl/fl^ and *Trim33*^fl/fl^ Cre-ER^T2^ CDPs over time were traced in 4-OHT-supplemented cultures, and a significant decrease in the survival of *Trim33*^fl/fl^ Cre-ER^T2^ cultures occurred before the decrease in the IRF8 level (Fig. S14). Therefore, TRIM33 maintained DC lineage survival through an IRF8-independent mechanism. We then further examined the effect of TRIM33 on the survival of downstream differentiated DCs. Indeed, we observed that DCs that differentiated from TRIM33-deficient pre-DCs also failed to persist in prolonged culture (Fig. S15A, B). More importantly, when *Trim33* was deleted at a later stage of DC development in *Trim33*^fl/fl^ *Itgax-*Cre mice, the RNA-seq-detected expression of critical DC differentiation-related genes was not altered in cKO DCs (Fig. S15C), suggesting that a failure of cell maintenance, rather than defective differentiation, was the major cause of reduced DC numbers in *Trim33*^fl/fl^ *Itgax-*Cre mice.

**Fig. 6:**
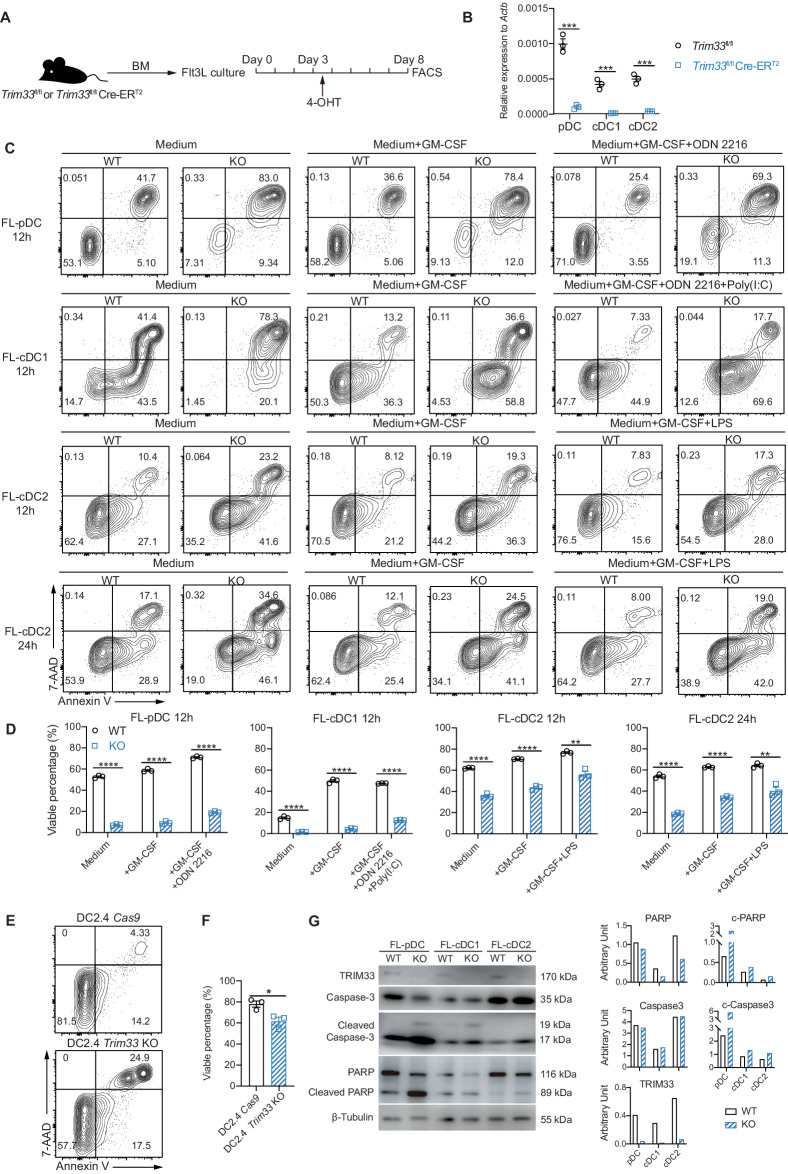
TRIM33 deficiency resulted in accelerated DC apoptosis. **A** Schematic diagram of the generation of DCs from WT or *Trim33* KO DCs in Flt3L cultures. BM cells from *Trim33*^fl/fl^ and *Trim33*^fl/fl^ Cre-ER^T2^ mice were cultured with 200 ng/mL Flt3L, and 1 µM 4-OHT was added on Days 3–4. FL-DCs were FACS-purified on Day 8. **B** qPCR analysis of *Trim33* expression in FL-DCs of the indicated genotypes, *n* = 3. **C**, **D** Survival analysis of cultured WT (*Trim33*^fl/fl^) and KO (*Trim33*^fl/fl^ Cre-ER^T2^) FL-DCs. A total of 5$\times$10^4^ FACS-purified FL-DCs/well were cultured with or without the indicated supplements (20 ng/ml GM-CSF, 1 µM ODN 2216, 100 µg/mL poly(I:C), or 100 ng/mL LPS) for 12 h or 24 h before detection. The numbers in quadrants indicate percentages. **D** The percentages of viable (Annexin V^–^7-AAD^–^) FL-DCs after culture, *n* = 3. **E**, **F** Survival analysis of DC2.4 cells of the indicated genotypes. A total of 2.5$\times$10^5^ DC2.4 or DC2.4 *Trim33* KO cells/well were seeded in 24-well plates and cultured for 48 h before detection. **F** The percentages of viable (Annexin V^–^7-AAD^–^) cells after culture are plotted as the means of 3 independent experiments. **G** Western blot analysis of Caspase-3 and PARP in FL-DCs cultured for 12 h. Approximately 1$\times$10^5^ cells/sample were loaded. The relative band intensities are shown. Bars represent the means ± SEMs. The data are representative of 3 independent experiments. Statistical significance was determined using an unpaired two-tailed Student’s *t* test. Ns: not significant, *P < 0.05, **P < 0.01, ***P < 0.001, and ****P < 0.0001

The viability of purified WT and TRIM33-deficient Flt3L-induced DC subsets (FL-DCs) (Fig. 6A, B) was measured by performing Annexin V and 7-AAD staining to further test whether TRIM33 regulates DC survival. In addition to the known rapid death of DCs in culture in vitro [71, 72], we found that all 3 subsets of FL-DCs exhibited accelerated cell death upon *Trim33* deletion (Fig. 6C, D), with or without granulocyte-macrophage colony stimulating factor (GM-CSF) and Toll-like receptor agonists, factors known to influence DC survival. Consistently, upon *Trim33* deletion, DC2.4 [73], a well-characterized DC cell line, also exhibited impaired cell survival (Fig. 6E, F). A greater proportion of Annexin V^+^7-AAD^–^ early apoptotic *Trim33*-deleted FL-cDCs indicated cell death by apoptosis (Fig. 6C). Moreover, increased cleavage of both Caspase-3 and PARP was detected in all 3 subsets of TRIM33-deficient FL-DCs, indicating that *Trim33* deletion resulted in enhanced activation of the apoptosis pathway (Fig. 6G). Taken together, these findings suggest that TRIM33 acts as a suppressor of DC apoptosis.

**Fig. 7:**
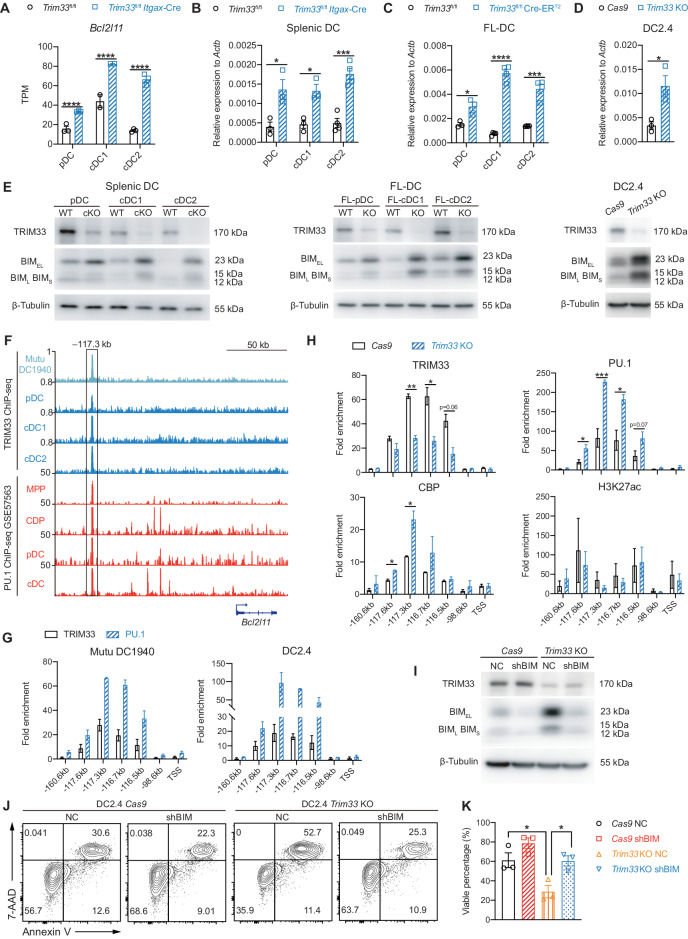
TRIM33 suppressed *Bcl2l11* transcription through enhancer deactivation. **A** RNA-seq detected *Bcl2l11* TPM in WT and cKO splenic DCs. *N* = 2-3, ****Q < 0.0001. qPCR analysis of *Bcl2l11* mRNA levels in (**B**) splenic DCs, (**C**) FL-DCs and (**D**) DC2.4 cells of the indicated genotypes. *N* = 3–4. **E** Western blot detection of BIM levels in splenic DCs, FL-DCs and DC2.4 cells of the indicated genotypes. **F** ChIP-seq tracks displaying TRIM33 and PU.1 (GSE57563) binding at the *Bcl2l11*-117.3 kb region in DCs and progenitors. The TRIM33-immunoprecipitated tracks are representative of 2 biological replicates. The Y-axis represents counts per million mapped reads (TRIM33-ChIP) or sequencing depth-normalized read counts (PU.1-ChIP). **G**, **H** ChIP‒qPCR detection of TRIM33, PU.1, CBP and H3K27ac intensity at the *Bcl2l11* locus in the indicated cell lines. Two to four biological replicates are plotted. The fold enrichment versus the isotype control IgG is presented. **I** *Bcl2l11* knockdown efficiency in DC2.4 cell lines. NC negative control, shBIM shRNA knockdown of *Bcl2l11*. **J**, **K** Survival analysis of DC2.4 cells of the indicated genotypes. **K** Percentages of viable cells (annexin V^–^7-AAD^–^) plotted using the means of 3 independent experiments. The data are representative of 3 independent experiments. Bars represent the means ± SEMs. Statistical significance was determined using unpaired two-tailed Student’s *t* tests (**B**–**D**, **H**) or multiple *t* tests (**K**). *P < 0.05, **P < 0.01, ***P < 0.001, and ****P < 0.0001

We analyzed the RNA-seq data again to explore how TRIM33 suppressed DC apoptosis, and the results revealed that the 3 subsets of TRIM33-deficient DCs exhibited no common downregulation of antiapoptotic genes, whereas the only upregulated proapoptotic gene in all DC subsets was *Bcl2l11* (Fig. S15D–F, Table S5, Fig. 7A). Increased *Bcl2l11* expression at both the mRNA and protein levels was found in all subsets of TRIM33-deficient splenic DCs, FL-DCs, and DC2.4 cells (Fig. 7B–E). Therefore, TRIM33 prevents DC apoptosis by suppressing *Bcl2l11* expression.

Given our previous results of TRIM33 and PU.1 colocalization at the -117.3 kb enhancer of *Bcl2l11* in CDPs (Fig. 5K), we further tested whether this colocalization also occurred in DCs. By performing ChIP-seq and ChIP‒qPCR analyses, we identified simultaneous TRIM33 and PU.1 occupancy at the reported -117.3 kb *Bcl2l11* enhancer but not at other sites in DC2.4 cells, Mutu DC1940 cells, and all subsets of primary DCs (Fig. 7F, G). The binding of PU.1 to such sites in MPPs was also revealed by investigating published data [65]. PU.1 activates transcription by recruiting CREB-binding protein (CBP) to bridge the basal transcription machinery and promote regional H3K27ac intensity [74, 75]. We found that in TRIM33-deficient DC2.4 cells, although the H3K27ac intensity was unaltered, the localization of PU.1 and CBP at the -117.3 kb region of the *Bcl2l11* enhancer site was enhanced (Fig. 7H). Therefore, TRIM33 occupancy at the -117.3 kb *Bcl2l11* enhancer in DCs antagonized PU.1-directed transcription of *Bcl2l11* and maintained DC survival. We also verified that efficient knockdown of *Bcl2l11* by an shRNA restored the survival of *Trim33* KO DC2.4 cells (Fig. 7I–K). Hence, TRIM33 likely suppresses DC apoptosis by repressing *Bcl2l11* transcription through antagonizing PU.1-induced *Bcl2l11* expression.

### TRIM33 regulated DC development and maintenance by modulating *Irf8* and *Bcl2l11* transcription

**Fig. 8:**
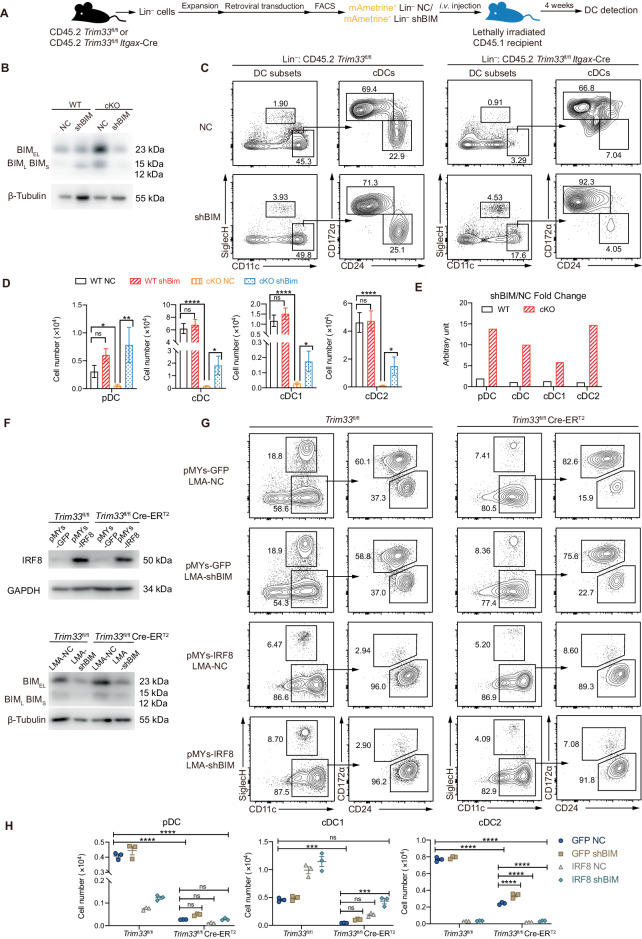
*Bcl2l11* knockdown and *Irf8* overexpression simultaneously restored cDC1 generation from TRIM33-deficient BM Lin^–^ precursors. **A** Scheme of the Lin^–^ cell in vivo reconstitution experiment. Lin^–^ cells were isolated from *Trim33*^fl/fl^ (WT) or *Trim33*^fl/fl^ *Itgax-*Cre (cKO) BM, expanded with Flt3L and SCF, transduced with shRNA constructs, and *i.v*. injected into recipients. **B** *Bcl2l11* knockdown efficiency in transduced mAmetrine^+^ WT and cKO Lin^–^ cells. **C–E** Flow cytometry analysis of splenic DC populations in the indicated Lin^–^ reconstituted mice. Pregate: viable, CD45.2^+^mAmetrine^+^ cells. The numbers on the plots indicate percentages. **D** Numbers of CD45.2^+^mAmetrine^+^ splenic DCs per animal from the indicated mice. **E** Fold increase in the number of each DC population of the indicated genotypes after *Bcl2l11* knockdown. *N* = 6–10. **F** *Irf8* overexpression and *Bcl2l11* knockdown efficiency in BM Lin^–^ cells. BM Lin^–^ cells of the indicated genotypes were cotransduced with overexpression and shRNA vectors as previously described. **G**, **H** Flow cytometry analysis of DCs generated from Lin^–^ BM cells transduced with the indicated construct pairs by Flt3L culture. Pregate: viable, CD45.2^+^GFP^+^mAmetrine^+^ cells. **H** Numbers of DCs generated per 1$\times$10^4^ input Lin^–^ cells. *N* = 3. Bars represent the means ± SEMs. The data are representative of 2–3 independent experiments. The data were analyzed using unpaired two-tailed multiple *t* tests (**D**) or two-way ANOVA followed by Bonferroni’s multiple comparison test (**H**). Ns: nonsignificant, *P < 0.05, **P < 0.01, ***P < 0.001, and ****P < 0.0001

BM CD45.2^+^ Lin^–^ precursors were isolated from *Trim33*^fl/fl^ and *Trim33*^fl/fl^ *Itgax-*Cre mice, transduced with the mAmetrine fluorescent protein-expressing negative control (NC) or shBIM vector, and allowed to restore DC homeostasis in lethally irradiated recipients to further validate whether TRIM33 suppressed *Bcl2l11* transcription to support DC maintenance in vivo (Fig. 8A). The knockdown of *Bcl2l11* in mAmetrine^+^ Lin^–^ cells was efficient (Fig. 8B), and in vivo differentiated CD45.2^+^ DCs retained vector expression upon analysis (Fig. S16A). We found that NC- and shBIM-transduced WT Lin^–^ cells generated all three subsets of splenic DCs in comparable numbers and proportions (Fig. 8C–E), in agreement with a previous report in which the DC numbers and percentages of *Bcl2l11*^–/–^ mice were comparable to those of WT mice [30]. In contrast, *Bcl2l11* knockdown significantly rescued the numbers of all DC subsets derived from CD45.2^+^ cKO Lin^-^ cells by 6-15-fold (Fig. 8C–E). No significant differences in the numbers of total CD45.2^+^ splenocytes in any of the experimental groups were observed (Fig. S16B). Thus, these data further show that TRIM33 maintains DC homeostasis by suppressing *Bcl2l11* expression.

Because we showed that TRIM33 was required for both promoting *Irf8* expression and tuning the PU.1-dependent transcriptome during DC development, as exemplified by the repression of *Bcl2l11*, we speculated that both functions of TRIM33 contributed to DC differentiation and maintenance, especially for cDC1s, considering the major fate-specific role of IRF8. To determine whether this is the case, We simultaneously overexpressed *Irf8* and knocked down *Bcl2l11* in *Trim33*^fl/fl^ or *Trim33*^fl/fl^ Cre-ER^T2^ BM Lin^–^ cells and measured their DC generation capacity to analyze our hypothesis. Notably, *Bcl2l11* knockdown may prevent an underestimation of the effects of *Irf8* overexpression; with *Bcl2l11* knockdown, any cell whose generation was rescued in culture may survive to the day of detection. The *Irf8* overexpression alone group and the *Bcl2l11* knockdown alone group were used as controls. Even with efficient overexpression or knockdown (Fig. 8F), *Irf8* overexpression or *Bcl2l11* knockdown alone was unable to significantly restore DC generation from TRIM33-deficient BM Lin^–^ cells (Fig. 8G, H), probably resulting from the death of the generated cells and developmental impairment, respectively. Nevertheless, *Irf8* overexpression combined with *Bcl2l11* knockdown significantly restored cDC1 production from TRIM33-deficient BM Lin^–^ precursors (Fig. 8H), further revealing the additive role of these TRIM33 targets in DC homeostasis. Taken together, these results further illustrate that TRIM33 promotes *Irf8* expression for cDC1 differentiation and suppresses PU.1-dependent *Bcl2l11* expression for DC survival and maintenance.

## Discussion

IRF8 is known as the key TF for cDC1 lineage specification. In this study, we identified TRIM33 as a novel regulator of IRF8 expression. TRIM33 deficiency significantly reduced the expression of *Irf8* and other DC-associated genes in DC progenitors, leading to a profound deficiency in DC differentiation. A mechanistic study revealed that TRIM33 was directly engaged at the gene body and the superenhancer of *Irf8*, possibly within complexes that modulate chromatin accessibility and transcription. In particular, by interacting with CDK9, the catalytic subunit of P-TEFb, TRIM33 promoted S2 Pol II localization and subsequent transcription of the *Irf8* gene, which facilitated *Irf8* expression and cDC1 differentiation. When the survival of all cells generated in culture was ensured by *Bcl2l11* inhibition, *Irf8* overexpression was able to reverse the deficiency of cDC1 development from TRIM33-ablated Lin^–^ precursors. Furthermore, we identified the –147 kb, –172 kb, and –197 kb sites as potential candidates for previously unrecognized *Irf8* enhancer regions, although whether these regions can control *Irf8* expression and DC development and whether they can bind to other important TFs require future exploration.

In addition to regulating *Irf8* expression and cDC1 fate specification, we found that TRIM33 was also crucial for tuning the PU.1-dependent transcriptome. Kusy et al. first reported that TRIM33 antagonizes PU.1-directed transcription by colocalization to repress exaggerated myelomonocytic expansion and verified this mechanism for 3 genes, *p15*^*INK4b*^, *Egr2*, and *Fcgr2b* [37]. With cell lines and bone marrow-derived macrophages, the same group further reported that TRIM33 and PU.1 colocalize at multiple sites in macrophages [43] and confirmed that TRIM33 antagonizes PU.1-directed transcription at distant *cis* elements of *Ifnb1* and *Atp1b3* [44, 76]. Wang et al. reported that TRIM33 antagonization of PU.1 at the -117.3 kb *Bcl2l11* enhancer is required for B-ALL cell survival [40]. In this study, we identified for the first time that TRIM33 also collaborated with PU.1 in DC fate determination and that numerous PU.1-dependent genes in DC progenitors were subjected to TRIM33 modulation through its occupancy at active enhancer sites. As proper tools for accurate annotation of distant enhancer-target gene relationships are lacking, we call for better resources in the future to fully elucidate the value of many of the shared peaks of TRIM33 and PU.1 in CDPs. Nevertheless, we were able to confirm that TRIM33 and PU.1 antagonize the –117.3 kb *Bcl2l11* enhancer and that they play a critical role in cell survival throughout the development and maintenance of the DC lineage. In this study, we showed for the first time that the survival of all subsets of DCs and DC progenitors required active suppression of *Bcl2l11* transcription by TRIM33, which prevented PU.1-programmed apoptosis under steady-state conditions. These findings should arouse interest, as the field has long been bothered by the short lifespan of DCs, while PU.1 was always considered a favorable master factor for DCs, or at least cDCs. Notably, the observed expansion of MPPs, CD115^–^ CDPs, and CLPs in TRIM33-deficient mice could have resulted from in vivo compensatory feedback responses to defective DC survival and a reduced pool size [77]. Interestingly, our findings, together with those of previous reports [36, 40, 41, 43], suggested that the antagonization of the PU.1-dependent -117.3 kb *Bcl2l11* enhancer by TRIM33 was required for the survival of DC, B cell, and macrophage lineages but not granulocyte or T-cell lineages.

The impaired pDC differentiation from progenitors and the exacerbated *Bcl2l11*-dependent cell death of DCs and their progenitors, especially CLPs, might collectively account for the marked reduction in pDCs in TRIM33-deficient mice. We observed significantly downregulated expression of pDC-associated genes in progenitors following TRIM33 deletion, which could partially result from the loss of TRIM33 antagonization of PU.1-mediated suppression of the pDC transcriptome, such as *Spib*. Chopin et al. showed that PU.1 favored a *Zfp366*-containing cDC transcription program over the pDC program and repressed pDC signatures in cDC determination [9]. Our results were consistent with these findings, showing that TRIM33 could suppress part of the PU.1-mediated transcriptional program to promote pDC development while collaborating with PU.1 for cDC determination. However, whether IRF8 functions as a cell-intrinsic factor required for pDC differentiation has been debated [10, 17, 78]. The *Irf8* overexpression system we used resulted in an exceedingly high IRF8 level, which strongly biased the precursors to cDC1 differentiation, hence reducing pDC production by WT progenitors. This finding may further support that IRF8 is not absolutely required for pDC specification and that an overly high level of IRF8 under certain circumstances may even inhibit pDC differentiation.

Regarding the role of TRIM33 in cDC2s, our current results confirmed the requirement of *Bcl2l11* suppression for the survival of cDC2s and their progenitors. Additionally, we illustrated that TRIM33 was required to facilitate the expression of PU.1-related cDC signature genes that promoted cDC2 development. Notably, in the motif analysis of TRIM33 binding sites, Krüppel-like factor (KLF) was identified; since KLF4 was found to be important for cDC2 cells, this possibility could be further explored. Interestingly, we also observed that elevated cDC1 production from WT progenitors with exceedingly high expression of *Irf8* occurred at the expense of cDC2s, consistent with the recent findings reported by Lanca et al. that IRF8 maintains the identity of terminally differentiated cDC1s and prevents reprogramming toward cDC2s [28]. However, in our animals, no expansion of the cDC2 subset was observed as a result of this mechanism. For *Trim33*^*fl/fl*^ *Itgax-*Cre mice, this outcome was more likely due to the unaltered expression of *Irf8* in DCs. Moreover, although the expression of *Irf8* was significantly reduced with Cre-ER^T2^-mediated early TRIM33 ablation in progenitors, *Bcl2l11* expression was enhanced in the same cells, which therefore led to impaired survival and differentiation of those progenitors to all DCs, including cDC2s. The differences we observed in cDC2 generation between in vivo and in vitro experiments, i.e., moderately reduced numbers of cDC2s in the spleens of TRIM33-deficient mice and TRIM33-deficient BM-reconstituted mice compared to the near absence of cDC2s in Flt3L cultures of TRIM33-deficient MPPs and CMPs, may be explained by the fact that the dying cDC2 progenitors could be continuously replenished by compensatory mechanisms in vivo, while cDC2s appeared to be relatively less sensitive to cell death signals than were pDCs and cDC1s [71, 72, 79]. In contrast, in the in vitro culture system, the dying DC progenitors could not be replenished, and the development of all DCs was severely impacted.

In summary, we reported for the first time that TRIM33 plays a crucial role in DC differentiation and homeostasis by promoting *Irf8* expression for cDC1 development and modulating PU.1 transcriptional functions, such as actively suppressing *Bcl2l11* expression in all DC subsets and their progenitors, to maintain cell survival. Given the unique role of cDC1s in antitumor immune responses, the identification of TRIM33 as a key regulator of cDC1 differentiation and DC survival may aid in the development of new strategies for DC-based immune modulations and therapies.

## Materials and methods

### Animals

*Trim33*^fl/fl^ mice on a C57BL/6 background were generated as previously reported [36, 46]. The floxed mice were crossed with Cre-ER^T2^ (Jax: 008463) mice or *Itgax-*Cre (Jax: 008068, gifted by Dr. Nan Shen, Shanghai Jiaotong University, China) to generate *Trim33*^fl/fl^ Cre-ER^T2^ and *Trim33*^fl/fl^ *Itgax-*Cre descendants. In vivo *Trim33* knockout by Cre recombinase was induced by an *i.p*. injection of 2 mg of tamoxifen (Sigma‒Aldrich, #T5648-1G) for 5 consecutive days, followed by additional maintenance for 7 days. Sex-matched 6- to 10-week-old mice were used in all experiments. Wild-type C57BL/6, CD45.1 (Jax: 002014), OT-I (Jax: 003831) and OT-II (Jax: 004194) mice were bred in house. All mice were maintained on a 12 L:12D cycle in a specific pathogen-free facility at Tsinghua University, Beijing, China. The experiments were conducted according to procedures approved by the Institutional Animal Care and Use Committee of Tsinghua University.

### Primary cell isolation

Tissues were cut, digested at room temperature for 30 min with 1 mg/mL collagenase III (Worthington Biochemical, #CLS-3) and 0.1 mg/mL DNase I (Roche, #10-104-159-001), and filtered through 70 µm strainers to obtain single-cell suspensions from the spleen, thymus, and lymph nodes. BM cells were flushed directly from the femurs and tibias. Erythrocytes were removed with homemade 0.168 M NH_4_Cl-containing red cell removal buffer (RCRB). Splenic DCs were enriched as previously described [80]. Briefly, light-density cells were collected after density gradient centrifugation with 1.077 g/cm^3^ Nycodenz (Axis-Shield), incubated with a homemade antibody cocktail against non-DC lineage markers (CD19, CD3ε, CD90, TER119, and Ly6G), and depleted of non-DC cells with BioMag immunomagnetic beads (Bangs Laboratories, #BM560). BM Lin^–^ precursors (CD2^–^ CD3^–^ CD8^–^ CD11b^–^ CD11c^–^ CD19^–^ B220^–^ TER119^–^ Ly6G^–^) were enriched in a similar manner; only 1.086 g/cm^3^ Nycodenz and a cocktail of anti-CD2, anti-CD3, anti-CD8, anti-B220, anti-CD11b, anti-Ly6G, and anti-TER119 antibodies were used.

### Cell culture

DC differentiation was induced in vitro by culturing 1.5-3$\times$10^6^/mL total BM cells or 3$\times$10^4^ FACS-purified progenitors plus 1.5$\times$10^5^ CD45.1 total BM feeders for 4–9 days in 200 ng/mL Flt3L (Peprotech, #96-250-31L-10)-supplemented HEPES-containing complete RPMI 1640 medium with 10% FBS, 100 U/mL penicillin‒streptomycin (Gibco, #15140122) and 50 µM 2-mercaptoethanol (Gibco, #21985023). Cre-dependent *Trim33* deletion was induced by adding 1 µM 4-hydroxytamoxifen (4-OHT, Sigma‒Aldrich, #H7904-5MG). For DC generation from genetically modified BM Lin^–^ cells, purified Lin^–^ cells were cultured at a density of 3-5$\times$10^5^/mL in medium supplemented with 200 ng/mL Flt3L (in vitro assays) or 100 ng/mL SCF (PeproTech, #250-03) + 30 ng/mL Flt3L (in vivo assays) before transduction with retroviruses. Lin^–^ cells were further cultured for 48-60 h, and 2-5$\times$10^4^ FACS-purified transduced cells were placed back into 200 µL of Flt3L and 1 µM 4-OHT-containing medium with 1.5$\times$10^5^ CD45.1 BM feeders and detected for DC generation after 5 additional days.

DC2.4, B16Flt3L melanoma, HEK293FT, Plat-E, BHK21, and Vero cells were cultured in DMEM (Gibco) supplemented with FBS and penicillin‒streptomycin. Mutu DC1940 cells were expanded in HEPES-containing IMDM (HyClone, #SH30228.01) supplemented with FBS, penicillin‒streptomycin and 2-mercaptoethanol.

For survival assays, sorted FL-DCs were cultured at 5$\times$10^4^ cells/well in complete RPMI 1640 medium for 12-24 h. The concentrations of the supplements used were 20 ng/mL GM-CSF (PeproTech, #315-03-100), 1 µM ODN 2216 (InvivoGen, #tlrl-2216-5), 100 µg/mL poly(I:C) (Sigma‒Aldrich, #P9582-5MG), and 100 ng/mL LPS (Sigma‒Aldrich, #L2630-10MG). A total of 2.5$\times$10^4^ cells/well of sorted progenitors was cultured in 1 µM 4-OHT-containing complete RPMI 1640 medium with or without 30 ng/mL Flt3L or 100 ng/mL SCF for 3–5 days. DC2.4 cells (2.5$\times$10^5^ cells/well) were seeded in 24-well plates and cultured for 48 h before survival was detected.

### BM chimeric mice

BM chimeric mice were generated by mixing 2$\times$10^6^ total BM cells from *Trim33*^fl/fl^ mice or *Trim33*^fl/fl^ Cre-ER^T2^ mice with 2$\times$10^5^ total wild-type CD45.1 BM cells, and these cells were *i.v*. injected into CD45.1 recipients lethally irradiated by two consecutive 5.5 Gy doses with 2 h of intermission. Mice were treated with tamoxifen 3 weeks after transplantation, as described above. For competitively reconstituted mice, 1$\times$10^6^ CD45.1 and 1$\times$10^6^ BM cells from CD45.2 *Trim33*^fl/fl^ or *Trim33*^fl/fl^ *Itgax-*Cre mice were *i.v*. injected into each recipient. For Lin^–^ reconstitution, 2$\times$10^5^ sorted mAmetrine^+^ Lin^–^ and 1.5$\times$10^5^ unfractionated RCRB-treated CD45.1 BM cells were *i.v*. injected into each recipient.

### Flow cytometry

Rat gamma globulin (Jackson, #012-000-002) or a homemade anti-CD16/32 antibody was used to minimize nonspecific staining. The cells were stained with the fluorescent dye-conjugated antibodies listed in Table S6 at 4 °C for 30 min, washed, and resuspended in 3% FBS-containing homemade balanced salt buffer. The apoptosis analysis was performed using Annexin V reagents and 7-AAD provided by BioLegend (Table S6) according to the manufacturer’s instructions. A Foxp3/transcription factor staining buffer set (eBioscience, #00-5523-00) was used to fix and permeabilize the cells for intracellular staining. Flow cytometry data were acquired with a BD LSRFortessa instrument and analyzed with FlowJo. Sorting was performed on a BD AriaIII instrument. The yields of the sorted progenitors or precursors per mouse are listed in Table S7.

### Genetic modifications through viral transduction

A *Trim33*-targeting single guide RNA (sequence: 5’-GCGGCCACCCCCCCGTCGTC-3’) was cloned and inserted into the LentiCRISPRv2-U6-Cas9-P2A-mRFP1 vector to perform gene knockdown with CRISPR/Cas9. Lentiviruses were packaged with HEK 293FT cells. DC2.4 cells were spinoculated with lentiviruses and 8 µg/mL polybrene (Macgene, Beijing, China) in a centrifuge at 2200 rpm and 32 °C for 2 h. Transduced cells were FACS-purified and subcloned by limited dilution.

For *Irf8* overexpression, the murine *Irf8* coding sequence was cloned and inserted into the pMYs-IRES-GFP vector (Cell Biolabs, #RTV-021). A targeting shRNA sequence (sense: 5’-GACGAGTTCAACGAAACTTAC-3’) was cloned and inserted into the pMSCV-LTRmiR30-IRES-mAmetrine (LMA) vector to knockdown *Bcl2l11* with RNAi. Plat-E cells were transfected with the plasmids to package retroviruses. The retroviruses were spun onto RetroNectin (Takara, #T100B)-coated nontissue culture plates at 37 °C and 2000 $\times \,$*g* for 2 h, after which expanded Lin^–^ cells were added for incubation for 48-60 h. The transduced cells were then sorted using a cytometer.

### RNA extraction and quantitative real-time PCR (qPCR)

Total RNA from the sorted cells was extracted with TRIzol reagent (Invitrogen, #15596018) following the manufacturer’s protocol. PrimeScript™ RT master mix (Takara, #RR036A) was used to synthesize cDNA. qPCR was performed on a 7900HT fast real-time PCR instrument (Applied Biosystems) with PowerUp™ SYBR™ green master mix (Applied Biosystems, #A25742). The primers [40, 81] used in this study are listed in Table S8.

### Mass spectrometry

Lin^–^FLT3^+^ cells isolated from the BM of wild-type C57BL/6 mice were lysed in Western and IP lysis buffer (Beyotime, #P0013) supplemented with proteinase and phosphatase inhibitors on ice for 30 min. The lysates were incubated with an anti-TRIM33 antibody (Bethyl Laboratories, #A301-059A) or control IgG (Cell Signaling Technology, #2729) at 4 °C overnight before the immunoprecipitates were purified with Protein A + G magnetic beads (Millipore, #16-663). The samples were then subjected to SDS‒PAGE for mass spectrometry analysis at the Technology Center for Protein Sciences of Tsinghua University. The bands were excised from the gel, reduced with 5 mM DTT and alkylated with 11 mM iodoacetamide, followed by in-gel digestion with sequencing-grade modified trypsin at 37 °C overnight. The peptides were extracted twice with 0.1% trifluoroacetic acid in a 50% aqueous acetonitrile solution for 30 min and then dried in a SpeedVac. Peptides were redissolved in 20 μl of 0.1% trifluoroacetic acid, and 6 μl of extracted peptides were analyzed with an LTQ Velos LTQ mass spectrometer (Thermo Scientific). For the LC‒MS/MS analysis, the peptides were separated by 120 min of gradient elution at a flow rate of 0.30 µl/min with a Thermo-Dionex Ultimate 3000 HPLC system, which was directly interfaced with a Thermo Scientific Q Exactive mass spectrometer. The analytical column was a homemade fused silica capillary column (75 µm ID, 150 mm length; Upchurch, Oak Harbor, WA) packed with C-18 resin (300 Å, 5 µm, Varian, Lexington, MA). The mobile phase consisted of 0.1% formic acid, and mobile phase B consisted of 80% acetonitrile and 0.1% formic acid. The LTQ Velos mass spectrometer was operated in the data-dependent acquisition mode using Xcalibur 2.1.2 software, and a single full-scan mass spectrum was recorded in the Orbitrap (300-1800 m/z, 70,000 resolution) followed by 20 data-dependent MS/MS scans at 27% normalized collision energy (HCD). The MS/MS spectra from each LC‒MS/MS run were searched against the Mouse.fasta database from UniProt using an in-house Proteome Discoverer (Version PD1.4, Thermo Fisher Scientific, USA). The search criteria were as follows: full tryptic specificity was needed; two missed cleavages were allowed; carbamidomethylation was set as the fixed modification; oxidation was set as the variable modification; precursor ion mass tolerances were set at 20 ppm for all MS acquired in an Orbitrap mass analyzer; and the fragment ion mass tolerance was set at 0.02 Da for all MS2 spectra acquired. The peptide spectrum match (PSM) was considered correct when q was <1%. The peptide false discovery rate (FDR) was calculated using Percolator provided by PD and determined based on PSMs when searched against the reverse decoy database. The FDR was also set to 0.01 for protein identification. Only peptides assigned to a given protein group were considered unique. The peak areas of fragment ions were used to calculate the relative intensity of precursor ions for selected peptides. The results were further filtered by unique peptides ≥ 2, score (anti-TRIM33-pulled) ≥ 2, and area (anti-TRIM33-pulled/IgG-pulled)>1.5 to generate 236 proteins considered authentic TRIM33-interacting molecules (Table S2). The R packages clusterProfiler 4.8.1 and enrichplot 1.20.0 [82] were used for GO biological process enrichment (Q < 0.05) and further analyses.

### Coimmunoprecipitation and Western blotting

Coimmunoprecipitation samples were acquired from Mutu DC1940 cells in an identical manner as described for mass spectrometry, but only the anti-CDK9 antibody (Abcam, #ab239364) was used along with the anti-TRIM33 antibody and IgG. For Western blotting, the samples were denatured, subjected to SDS‒PAGE, and transferred to activated PVDF membranes. After blocking, the membranes were incubated with the primary antibodies listed in Table S6 overnight at 4 °C. After the membranes were washed and incubated with HRP-conjugated secondary antibodies, SuperSignal™ West Pico PLUS chemiluminescent substrate (Thermo Fisher Scientific, #34578) was used for imaging with an Amersham Imager 600 System (GE).

### RNA sequencing

Wild-type or TRIM33-deficient progenitors and splenic DCs were sorted from 6- to 8-week-old mice. Total RNA was extracted with TRIzol. BGI-Tech (Shenzhen, China) constructed the mRNA library, performed sequencing, and analyzed the data. DNA nanoballs generated by rolling circle replication were read with the BGISEQ-500 platform (Shenzhen, China) to generate single-end 50-base reads. The raw reads were filtered with SOAPnuke v1.5.2 to remove adapter-containing, low-quality, or >10% unknown base-containing reads. Clean reads were mapped to the mm10 reference genome by HISAT2 v2.0.4 [83] to generate BAM files. For the gene expression analysis, clean reads were mapped to a reference using Bowtie2 v2.2.5 [84], and gene expression levels were calculated with RSEM v1.2.12 [85]. DEGs were detected for MPPs and CD115^–^CDPs using DEGseq v1.48.0 (log2|FC | ≥ 1, Q-value ≤ 0.001) [86], for CLPs using DEseq2 v.1.4.5 (log2|FC | ≥ 1, Q-value ≤ 0.05) [87], and for DCs using DEseq2 (log2|FC | ≥ 0.5, Q-value ≤ 0.01).

### CUT&Tag, chromatin immunoprecipitation assays, sequencing, and data analysis

Sorted wild-type CD11c^–^CD127^–^CD117^int^FLT3^+^ CDPs were subjected to a CUT&Tag assay and library generation from 1$\times$10^5^ cells/sample with the Hyperactive In-Situ ChIP Library Prep Kit (Vazyme) following the manufacturer’s instructions, as described previously [88]. The antibodies used in this study are listed in Table S6. Sample quality verification, sequencing of PE150 reads on the HiSeq X platform, and raw read processing were performed by Annoroad Gene Technology (Beijing, China). Sorted BM Lin^–^ cells from tamoxifen-treated *Trim33*^fl/fl^ or *Trim33*^fl/fl^ Cre-ER^T2^ mice and cultured DC2.4 cells or Mutu DC1940 cells were used for ChIP. Large numbers of primary DCs for ChIP-seq sample preparation (Fig. 7F) were acquired after 2$\times$10^6^ B16Flt3L cells were injected subcutaneously into wild-type male C57BL/6 mice to increase DC numbers [89]. Splenic cDCs and BM pDCs were sorted 2 weeks after inoculation as described. For ChIP-seq or ChIP‒qPCR, 2$\times$10^7^ cultured or sorted cells/sample were prepared with the ChIP-IT^®^ Express Enzymatic Shearing Kit (Active Motif, #53035) after crosslinking the cells with 1% formaldehyde at room temperature for 10 min and quenching with 0.125 M glycine. The cells were digested, sonicated, and enzyme-sheared to obtain fragmented chromatin, which was incubated with antibodies (Table S6) or IgG at 4 °C overnight. The immunoprecipitated chromatin was purified with Magna ChIP™ Protein A + G magnetic beads (Millipore, #16-663), decrosslinked, and extracted with a MinElute PCR purification kit (Qiagen, #28004). Sample quality was checked on a D1000 ScreenTape system (Agilent, Santa Clara, CA) by electrophoresis, and samples with fragment sizes between 150 and 1000 bp and major peaks between 150 and 300 bp were considered appropriate for further experiments. For samples sent for ChIP-seq, aliquots were retained for future ChIP‒qPCR verification. qPCR was performed as described above. The primers used for the *Irf8* locus and the primers used for the *Bcl2l11* upstream region [40] can be found in Table S8. For ChIP-seq, libraries were constructed following standard Illumina protocols and sequenced on the Illumina NovoSeq 6000 platform to generate paired-end 150 bp reads. Raw reads of sequencing data in FASTQ format were processed through fastp (v0.19.11) [90] or Trimmomatic (v0.40). BWA-MEM (v0.7.12) or Bowtie2 (v2.2.5) was used for mapping reads onto the reference genome (mm10). BAM files were indexed with samtools (v.1.4.1), and bigwig files for visual inspection with Integrative Genomic Viewer were generated using deepTools (v.3.5.2) [91, 92]. Wig tracks of the mm9 build in the GSE57563 and GSE66899 datasets were lifted over to mm10 with CrossMap (v.0.3.3) for comparison with the tracks generated in this study. Peaks were called with MACS2 (v.2.2.7.1) [93]: TRIM33 and CDK9 CUT&Tag—callpeak -m 2 50 q 0.01; S2PolII CUT&Tag—callpeak -m 2 50 q 0.01 –broad; ATAC-seq (GSE132240)—bdgpeakcall -c 2 -l 300 -g 150; H3K27ac ChIP-seq (GSE132239)—bdgbroadcall -c 2 -l 300 -g 150 -G 1200; PU.1 ChIP-seq (GSE57563)—bdgpeakcall-c 100-l 300-g 30; IRF8 ChIP-seq (GSE66899)—bdgpeakcall-c 5-l 300-g 30. Bedgraphs of PU.1 and IRF8 were preanalyzed for the optimum cutoffs to match the peak numbers in previous reports. Intervene (v0.6.4) [94] was used to intersect genomic regions. A heatmap of the binding distribution was generated with deepTools. The R package ChIPseeker (v.1.36.0) was utilized for peak annotation and the feature/distribution analysis, and clusterProfiler (v.4.8.1) was used for GO enrichment with a Q value cutoff set to 0.05 [82, 95]. The motif enrichment analysis was performed with MEME-ChIP under the default settings [96]. TSS ± 2 kb regions of RNA-seq-detected DEGs were subjected to a TF enrichment analysis with ChIP-Atlas [97]. The enhancer analysis was performed with SEA 3.0 [66] and Enhancer Atlas 2.0 [67].

### Enzyme-linked immunosorbent assay (ELISA), in vivo antigen presentation assay, and LCMV infection model

The procedures are detailed in the Supplementary Materials.

### Quantification and statistical analysis

All statistical analyses for non-RNA-seq data were performed with GraphPad Prism 8.3. The exact sample size (N) and statistical test results for each experiment are provided in the figure legends. Each presented experiment was representative of at least 2-3 independent repetitions to ensure data reproducibility. No data were excluded. A replicate is technical for the ChIP‒qPCR experiments in Fig. 5 and biological for any other experiments. Bars represent the means ± SEMs. A *P* value less than 0.05 was considered significant.

### Supplementary information

Supplementary Material

Table S1

Table S2

Table S3

Table S4

Table S5

Table S6

Table S8

Uncropped Western blot gels

## Data Availability

The generated datasets have been deposited in Gene Expression Omnibus and are publicly available as of the date of publication under the accession numbers GSE194246 (progenitor RNA-seq), GSE193183 (DC RNA-seq), GSE255136 (CDP TRIM33, CDK9, S2 Pol II CUT&Tag), GSE193918 (Mutu DC1940 ChIP-seq), and GSE196586 (primary DC ChIP-seq). Supplementary information is available at the *Cellular & Molecular Immunology* website.
