# Supplementary material for: TRIM33 plays a critical role in regulating dendritic cell differentiation and homeostasis by modulating Irf8 and Bcl2l11 transcription
Source: Cell Mol Immunol. 2024 May 31;21(7):752–69. doi: 10.1038/s41423-024-01179-1 (PMC11214632; doi:10.1038/s41423-024-01179-1)
Supplement: Supplementary file 1 — Supplementary Material [file 41423_2024_1179_MOESM1_ESM.pdf]

LIST OF SUPPLEMENTARY MATERIALS

Supplementary Materials and Methods (within this PDF)
Fig. S1. *Trim33* expression and CD11c$^+$-conditioned *Trim33* deletion. (within this PDF)
Fig. S2. Additional DC profile of mice with CD11c$^+$-conditioned TRIM33 deficiency. (within this PDF)
Fig. S3. Defective antigen-specific T cell priming and anti-LCMV immunity of mice with CD11c$^+$-conditioned TRIM33 deficiency. (within this PDF)
Fig. S4. Additional DC profile of Tamoxifen-inducible Cre-mediated *Trim33* deletion. (within this PDF)
Fig. S5. Immune cell profile of CD45.1 WT BM-reconstituted chimeras. (within this PDF)
Fig. S6. Analysis of newly identified DC subsets in indicated genotypes. (within this PDF)
Fig. S7. Flow cytometric analysis of DC subsets generated in Flt3L cultures at different time points. (within this PDF)
Fig. S8. Post-sort validation of purified DC progenitors. (within this PDF)
Fig. S9. Survival analysis of TRIM33-deficient progenitors. (within this PDF)
Fig. S10. RNA-seq analysis detected DEGs in WT and TRIM33-deficient DC progenitors. (within this PDF)
Fig. S11. Analysis of TRIM33-interacting proteins detected by co-IP/MS. (within this PDF)
Fig. S12. Additional profile of TRIM33 genome-wide binding sites in CDP. (within this PDF)
Fig. S13. DC generation from *Irf8*-overexpressing TRIM33-deficient BM Lin$^-$ cells in Flt3L cultures. (within this PDF)
Fig. S14. Time course analysis of CDP cultures *in vitro*. (within this PDF)
Fig. S15. Transcriptome analysis of TRIM33-deficient terminally differentiated DCs with defective maintenance. (within this PDF)
Fig. S16. Additional properties of CD45.2$^+$ cells reconstituted from BM Lin$^-$ cells *in vivo*. (within this PDF)
Table S1. RNA-seq analysis detected genes downregulated in more than one DC progenitor populations upon TRIM33 deficiency (.xls file).
Table S2. Co-IP/MS identified TRIM33-interacting proteins in Lin$^-$FLT3$^+$ cells (.xls file).
Table S3. CDP genes under dual regulation of TRIM33 and PU.1 (.xls file).
Table S4. Shared genomic binding regions of TRIM33 and PU.1 in CDP (.xls file).
Table S5. RNA-seq analysis detected differentially expressed genes in WT and cKO splenic DCs (.xls file).
Table S6. Antibodies used in the study (.xls file).
Table S7. Yield of DC progenitor or precursor per mice by FACS. (within this PDF)
Table S8. Primers used for quantitative real-time PCRs (.xls file).

Supplementary Materials and Methods

Enzyme-Linked Immunosorbent Assay (ELISA)

The sandwich method of ELISA was used for serum IFN-α and IFN-γ concentration determination. For IFN-α, the rat RMMA-1 mAb #22100-1 (PBL) was used for capture, the rabbit serum pAb #32100-1 (PBL) was used for detection, and the Peroxidase-AffiniPure Goat Anti-Rabbit IgG (H+L) antibody (Jackson, #111-035-144) was used for chromogenic reaction. The Mouse IFN Alpha A recombinant protein (PBL, #12100-1) was used to prepare standard samples. For IFN-γ, the capture antibody AN-18 (eBioscience #14-7313), biotinylated detection antibody R4-6A2 (eBioscience, #13-7312), HRP Streptavidin (BioLegend, #405210) and standard protein (eBioscience, #39-8311) were used. The TMB Substrate Set (#421101, BioLegend) was used for spectrophotometric reading at 450 nm.

In vivo Antigen Presentation Assay

Splenic $CD8^+$ T cells of OT-I mice or $CD4^+$ T cells of OT-II mice were sorted, labeled with CFSE eBioscience, #65-0850-84), and *i.v.* injected to *Trim33*$^{fl/fl}$ and *Trim33*$^{fl/fl}$ *Itgax*-Cre mice (7 - 10 $\times$ 10^5 cells/mouse). The recipients received 2 subcutaneous injections of 50 μg soluble OVA or PBS of equal volume on each side of the inguinal region 24 h post transfer. Both inguinal draining lymph nodes were collected 48 h later, digested as described above, stained, and subjected to flow cytometry analysis. Proliferation of OVA-specific OT-I $CD8^+$ T cells ($CD3\varepsilon^+$ $CD8^+$ TCR $V\alpha2^+$ $CFSE^+$) or OT-II $CD4^+$ T cells ($CD3\varepsilon^+$ $CD4^+$ TCR $V\alpha2^+$ $CFSE^+$) was quantified by CFSE signal decay.

LCMV Infection Model

LCMV Armstrong was propagated on BHK21 cells and titrated by plaque assay on Vero cells (gifted by Dr. L. Zhang, Tsinghua University, China) as. To initiate acute LCMV infection, each mouse received *i.p.* inoculation of 2 $\times$ 10^5 plaque forming units. To obtain sera for ELISA assays, mice were anesthetized and bled from the orbital sinus 24 h or 120 h post infection. To measure the viral load in spleen, on day 7 post infection, 50 - 100 mg harvested tissue was homogenized in 1 mL cold TRIzol and subjected to further RNA extraction. The PrimeScript™ RT reagent Kit with gDNA Eraser (Takara, #RR047A) and viral gene-specific primers NP2-R and GP-R (Table S8) were used to reverse transcribe cDNA from 1 μg total RNA. Viral NP and GP copy numbers were measured by qPCR as described above with primers listed in Table S8. Plaque assay-titrated standard samples were used as calculation references. The detection limit of viral load was 3 $\times$ 10^5 copies/g tissue.

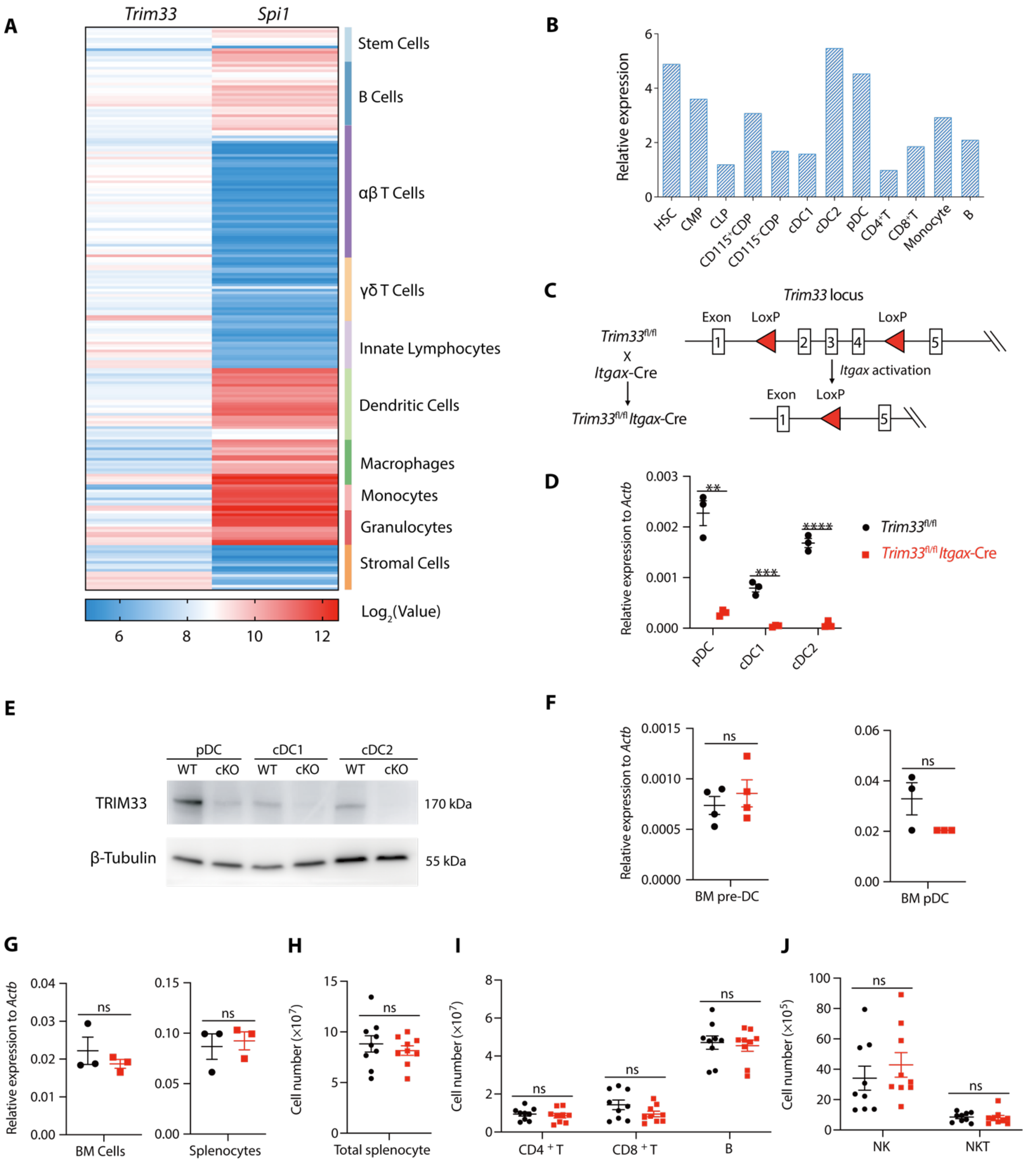

Fig. S1. *Trim33* expression and CD11c^{+}-conditioned *Trim33* deletion.

(A) The expression of *Trim33* and *Spi1*[PU.1] among immune cell types. Heatmap is plotted with Immgen microarray V1 data.

(B) *Trim33* expression by DCs, DC progenitors, and other immune cells from wild-type C57BL/6 mice. *Trim33* expression is detected by qPCR and data was normalized to mRNA level in CD4^{+} T cells. *Gapdh* was used as internal reference.

(C) Breeding scheme of *Trim33*$^{fl/fl}$ *Itgax*-Cre mice.

(D) qPCR analysis of *Trim33* expression in sorted splenic DCs of *Trim33*$^{fl/fl}$ and *Trim33*$^{fl/fl}$ *Itgax*-Cre mice.

pDCs: CD11c^{+} SiglecH^{+}, cDC1s: CD11c^{+} SiglecH$^{–}$ MHC II^{+} CD24^{+} CD172α$^{–}$, cDC2s: CD11c^{+} SiglecH$^{–}$ MHC II^{+} CD24$^{–}$ CD172α^{+}. Means of 3 independent experiments were pooled and plotted. Each dot represents a sample acquired from 4-5 (*Trim33*$^{fl/fl}$) or 10 (*Trim33*$^{fl/fl}$ *Itgax*-Cre) mice.

(E) Western blot for TRIM33 levels in sorted splenic DCs of indicated genotypes. Cells were sorted using gating strategy described in (D). Plots are representative of 3 independent experiments.

(F) qPCR detection of *Trim33* expression in BM pDCs and pre-DCs of *Trim33*$^{fl/fl}$ and *Trim33*$^{fl/fl}$ *Itgax*-Cre mice. Means of 3-4 independent experiments were pooled and plotted. Cells were gated for sorting as in (F). Each dot represents a sample acquired from 4-5 mice.

(G) qPCR analysis of *Trim33* expression in bone marrow (BM) cells and splenocytes of *Trim33*$^{fl/fl}$ and *Trim33*$^{fl/fl}$ *Itgax*-Cre mice. Means of 3 independent experiments were pooled and plotted. Each dot represents a biological replicate.

(H-J) Flow cytometry-detected numbers of indicated cell types of *Trim33*$^{fl/fl}$ and *Trim33*$^{fl/fl}$ *Itgax*-Cre mice. Scatterplots show numbers of total splenocytes as well as splenic CD4^{+} T cells (CD19$^{–}$ CD3ε^{+} NK1.1$^{–}$ CD4^{+} CD8α$^{–}$), CD8^{+} T cells (CD19$^{–}$ CD3ε^{+} NK1.1$^{–}$ CD4$^{–}$ CD8α^{+}), B cells (CD19^{+}), NK cells (CD19$^{–}$ CD3ε$^{–}$ NK1.1^{+}) and NKT cells (CD19$^{–}$ CD3ε^{+} NK1.1^{+}). n = 9. Each dot represents individual mouse. Data are pooled over 2-3 independent experiments.

Error bars represent mean ± SEM. Absolute cell number per animal was shown. Statistical significance was determined by unpaired two-tailed Student's *t* test. ns: $P > 0.05$. ** $P < 0.01$, *** $P < 0.001$, **** $P < 0.0001$.

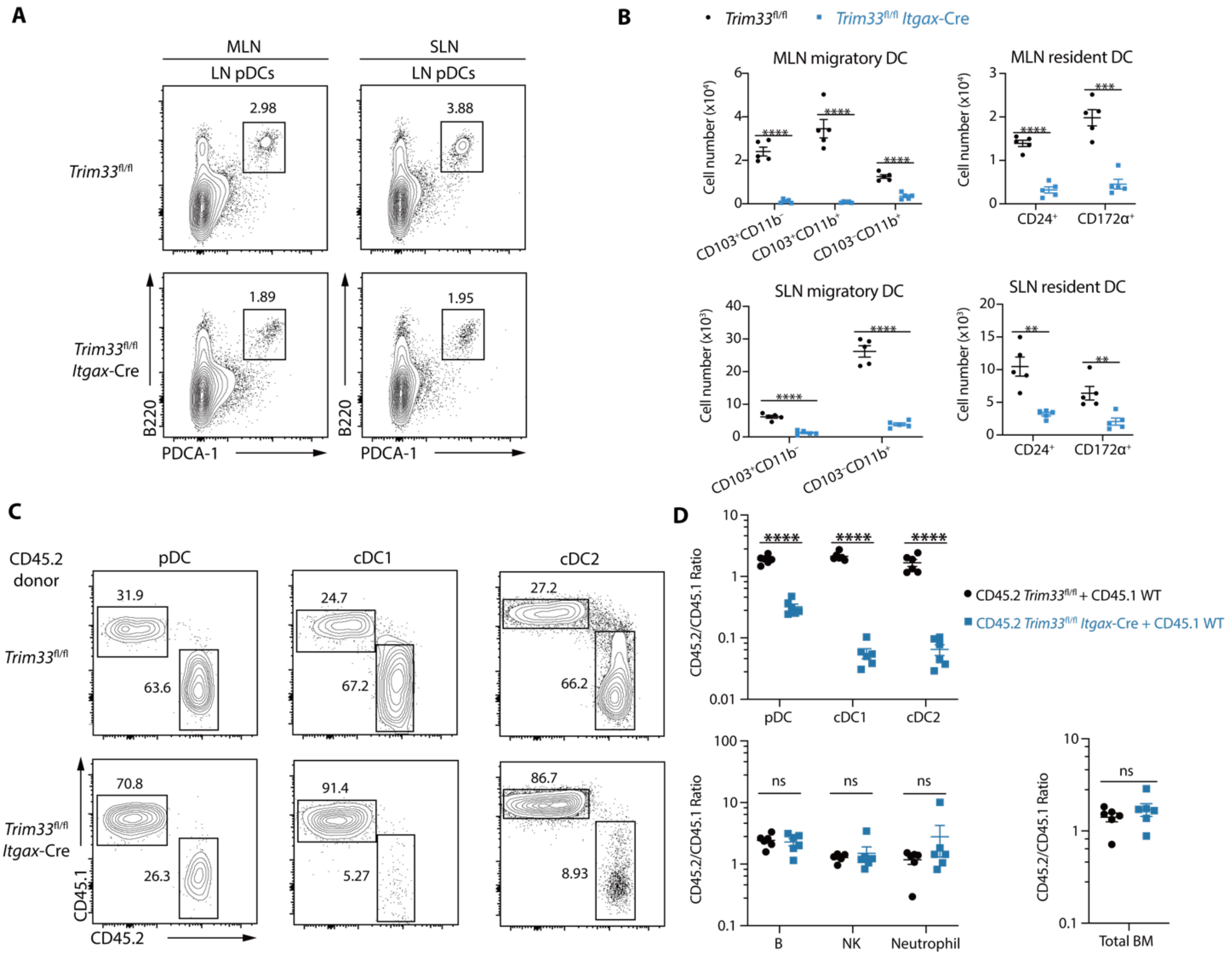

Fig. S2. Additional DC profile of mice with $CD11c^+$-conditioned TRIM33 deficiency.

(A and B) Flow cytometric analysis for DC populations in the mesenteric (n = 6) and skin-draining (n = 6) lymph nodes of *Trim33*$^{fl/fl}$ and *Trim33*$^{fl/fl}$ *Itgax*-Cre mice. MLN, mesenteric lymph node. SLN, subcutaneous lymph node. Numbers of $CD11c^+$ $MHC\text{-}II^{hi}$ mDC or $CD11c^{hi}$ $MHC\text{-}II^+$ rDC subpopulations of indicated organs and genotypes are plotted in (B).

(C) Representative flow cytometric plots showing CD45.1 and CD45.2 composition of splenic DCs of indicated competitively reconstituted chimeras. BM of CD45.2 *Trim33*$^{fl/fl}$ or *Trim33*$^{fl/fl}$ *Itgax*-Cre mice was mixed with WT CD45.1 BM at a 1:1 ratio. 1 $\times 10^6$ CD45.2 BM and 1 $\times 10^6$ CD45.1 BM was *i.v.* injected to each lethally irradiated CD45.1 recipient. Reconstituted cell populations were analyzed at least 3 weeks later. pDCs: $CD11c^+$ SiglecH, cDC1s: $CD11c^{hi}$ $SiglecH^-$ MHC II^+ $CD24^+$ $CD172\alpha^-$, cDC2s: $CD11c^{hi}$ $SiglecH^-$ MHC II^+ $CD24^-$ $CD172\alpha^+$. Numbers on plots indicate percentages of parent.

(D) Scatterplots showing CD45.2/CD45.1 ratios of total BM (n = 6), splenic DCs (n = 6) and other splenic immune cells (n = 6) of indicated chimeras. B cells: $CD19^+$, NK cells: $CD19^-$ $CD3\varepsilon^-$ $NK1.1^+$, neutrophils: $CD11b^+$ $Ly6G^{hi}$.

Data are representative of 2 - 5 independent experiments. Flow cytometry plots shown are pre-gated for viable cells. Splenic and thymic samples were pre-enriched for DCs before detection. Numbers adjacent to the plotted populations indicate percentages of parent. Dots represent absolute numbers of each individual mice. Error bars represent mean ± SEM. Statistical significance was determined by unpaired two-tailed Student's *t* test. ns: $P > 0.05$. ** $P < 0.05$, ** $P < 0.01$, *** $P < 0.001$, **** $P < 0.0001$.

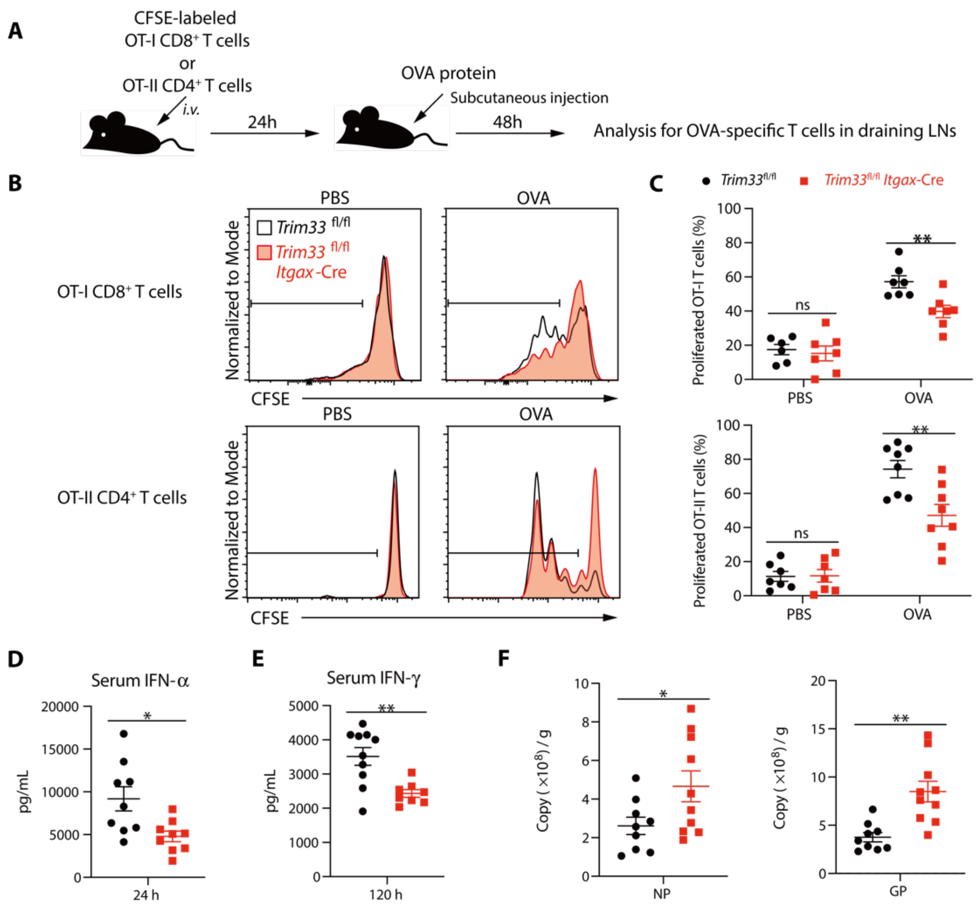

Fig. S3. Defective antigen-specific T cell priming and anti-LCMV immunity of mice with CD11c+-conditioned TRIM33 deficiency.

(A) Experimental design of antigen presentation assay *in vivo*. CFSE-labeled OT-I $CD8^+$ T cells (7×10^5/mice) or OT-II $CD4^+$ T cells (1×10^6/mice) were *i.v.* injected to *Trim33*$^{fl/fl}$ and *Trim33*$^{fl/fl}$ *Itgax*-Cre mice. Ovalbumin (OVA) protein (50 μg/side) or PBS was subcutaneously injected to the inguinal region of mice at both sides 24 h after transfer. Both inguinal lymph nodes of recipients were collected and analyzed 48 h after antigen stimulation.

(B, C) Flow cytometry analysis of OVA-specific T cell proliferation *in vivo*. Pre-gate: single, viable, $CD3\varepsilon^+$ TCR $V\alpha2^+$ $CFSE^+$ $CD8^+$ (OT-I subgroups, n = 6 - 7) or $CD4^+$ (OT-II subgroups, n = 7 - 8) cells. Scatterplots indicate proliferated percentages of transferred T cells. Dots represent individual mice.

(D, E) ELISA-detected WT and cKO serum concentration of indicated cytokines 24 h or 120 h post LCMV Armstrong infection. 2×10^5 plaque forming units were *i.p.* injected to elicit acute infection. Data are pooled over 3 independent experiments, n = 6 - 10. Dots represent individual mice.

(F) qPCR-determined splenic LCMV genome copy numbers in WT and cKO mice 7 days post infection (n = 8 - 10). NP, nucleoprotein; GP, glycoprotein. Method detection limit is 3×10^5 copies per gram tissue.

Error bars represent mean ± SEM. Statistical significance was determined by unpaired two-tailed Student's *t* test. Ns: non-significant, * $P < 0.05$, ** $P < 0.01$.

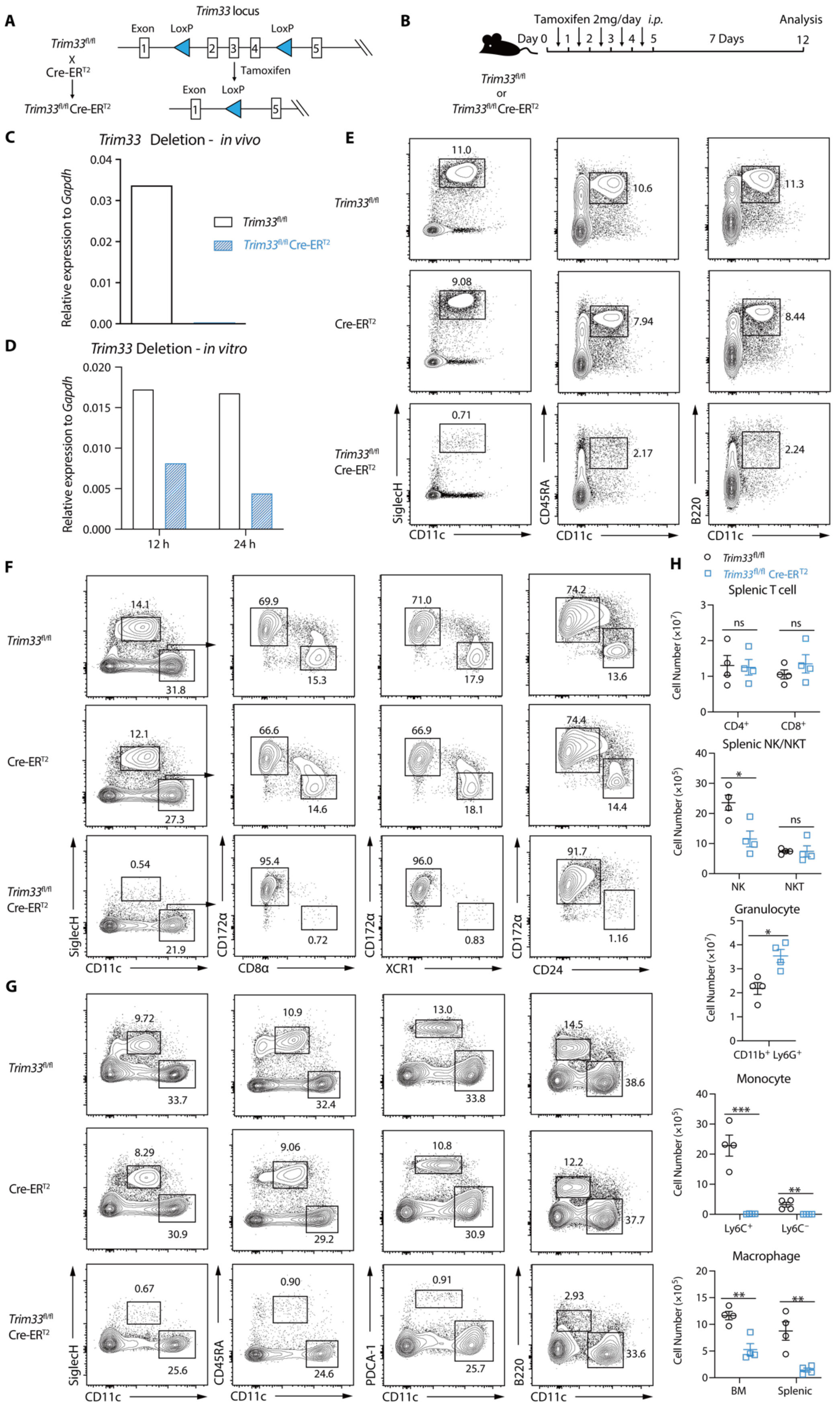

Fig. S4. Additional profile of Tamoxifen-inducible Cre-mediated *Trim33* deletion.

(A and B) Generation of TRIM33-deficient mice using Tamoxifen induced-Cre/LoxP system. Mice were *i.p.* injected with 2 mg tamoxifen for 5 consecutive days and analyzed after 7 additional days.
(C and D) Deletion efficiency of *Trim33* in BM Lin^{-}cells by Cre recombinase induction *in vivo* and *in vitro.* (C) indicates qPCR-detected *Trim33* mRNA level in BM Lin^{-} cells sorted from *Trim33*$^{fl/fl}$ and *Trim33*$^{fl/fl}$ Cre-ER^{T2} mice treated by *i.p.* injection of 2 mg Tamoxifen for 5 consecutive days and analyzed 7 days later. (D) shows qPCR-detected *Trim33* mRNA level in BM Lin^{-} cells cultured *in vitro* with 1 μM 4-OHT for indicated time.
(E) Flow cytometric analyses of BM pDC of Tamoxifen-treated mice of indicated genotypes. Cells pre-gated on viable, $CD11b^{-}$ $CD19^{-}$. Gating strategies for pDC using SiglecH, CD45RA and B220 with CD11c respectively are shown.
(F) Flow cytometric analyses of splenic DC populations of tamoxifen-treated mice in indicated genotypes. Viable cells were pre-gated. Gating strategies for cDC populations using CD8α, XCR1 and CD24 with CD172α respectively are shown.
(G) Flow cytometric analyses of splenic DC populations of tamoxifen-treated mice of indicated genotypes. The viable cells were pre-gated. Gating strategies for pDC using SiglecH, CD45RA, PDCA-1 and B220 with CD11c respectively are shown.
(H) Flow cytometric analyses of indicated immune cell types. Numbers of $CD4^{+}$ T cells ($CD19^{-}$ $CD3\varepsilon^{+}$ $NK1.1^{-}$ $CD4^{+}$ $CD8\alpha^{-}$), $CD8^{+}$ T cells ($CD19^{-}$ $CD3\varepsilon^{+}$ $NK1.1^{-}$ $CD4^{-}$ $CD8\alpha^{+}$), NK cells ($CD19^{-}$ $CD3\varepsilon^{-}$ $NK1.1^{+}$), NKT cells ($CD19^{-}$ $CD3\varepsilon^{+}$ $NK1.1^{+}$), neutrophils ($CD11b^{+}$ $Ly6G^{hi}$), macrophages ($CD11b^{+}$ $Ly6G^{-}$ $F4/80^{+}$), and monocytes ($CD11b^{+}$ $Ly6G^{-}$ $CD115^{+}$) were shown.
Representative plots of 3-5 biological repeats are shown. Absolute number per mice was plotted. Error bars represent mean ± SEM. Statistical significance was determined by unpaired two-tailed Student's *t* test. Ns: non-significant, * $P < 0.05$, ** $P < 0.01$, *** $P < 0.001$.

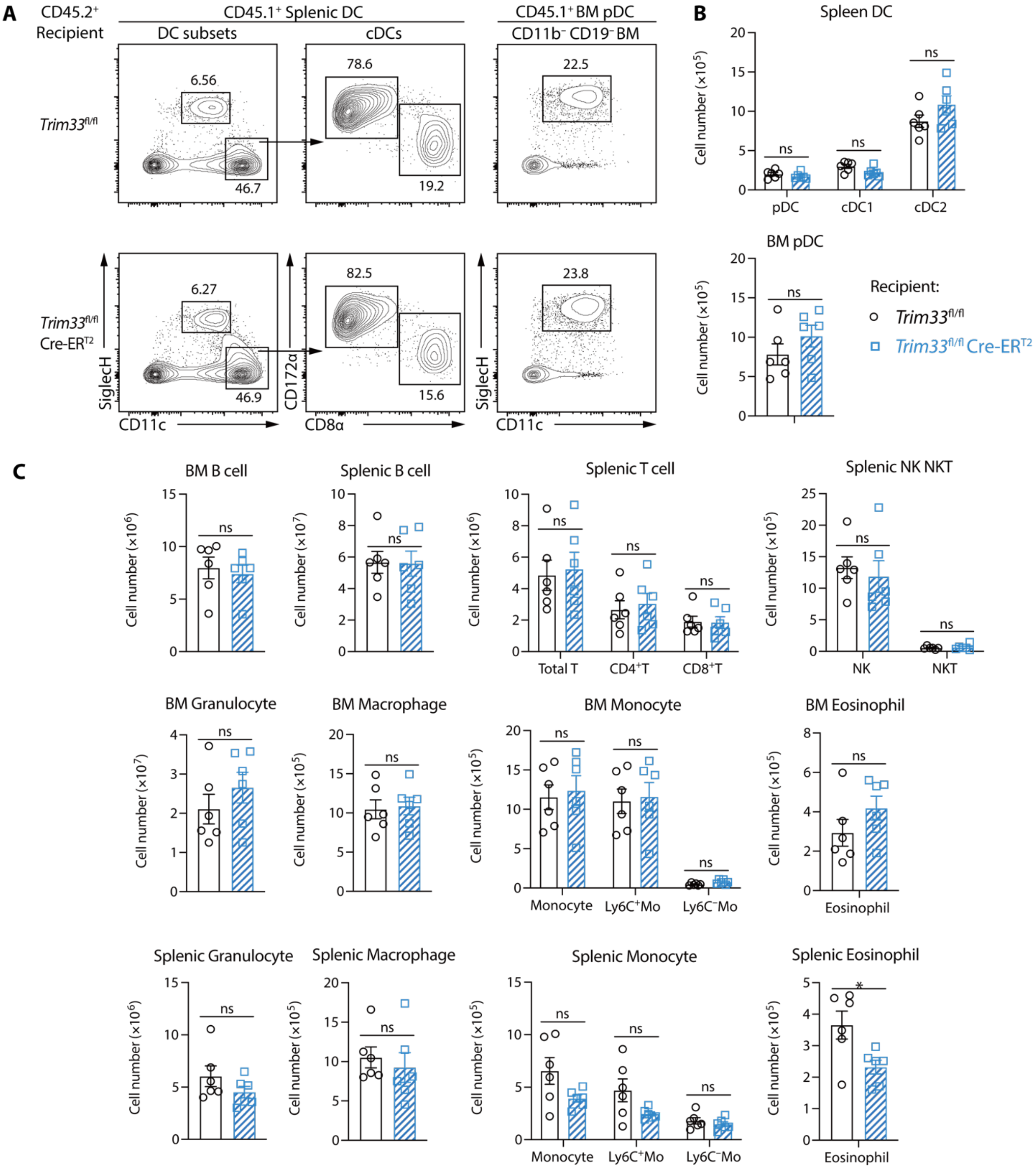

Fig. S5. Immune cell profile of CD45.1 WT BM-reconstituted chimeras.

(A-B) Flow cytometric analysis CD45.1 DC populations in indicated reconstituted chimeras. 2×10^6 BM cells of CD45.1 wild-type mice were injected to each lethally irradiated CD45.2 *Trim33*$^{fl/fl}$ or *Trim33*$^{fl/fl}$ Cre-ER^{T2} recipient. Mice were treated with Tamoxifen 3 weeks post reconstitution. Pre-gate: alive. Splenic DCs were pre-enriched. pDCs: CD11c$^+$ SiglecH, cDC1s: CD11c^{hi} SiglecH$^-$ MHC II$^+$ CD8α$^+$ CD172α$^-$, cDC2s: CD11c^{hi} SiglecH$^-$ MHC II$^+$ CD8α$^-$ CD172α$^+$. Numbers adjacent to gates indicate percentages of parent.

(C) Profiling of CD45.1 immune cells in indicated reconstituted chimeras. Numbers of B cells (CD19$^+$), total T cells (CD19$^-$ CD3ε$^+$ NK1.1$^-$), CD4$^+$ T cells (CD19$^-$ CD3ε$^+$ NK1.1$^-$ CD4$^+$ CD8α$^-$), CD8$^+$ T cells (CD19$^-$

$CD3\varepsilon^+$ $NK1.1^-$ $CD4^-$ $CD8\alpha^+$), NK cells ($CD19^-$ $CD3\varepsilon^-$ $NK1.1^+$), NKT cells ($CD19^-$ $CD3\varepsilon^+$ $NK1.1^+$), neutrophils ($CD11b^+$ $Ly6G^{hi}$), macrophages ($CD11b^+$ $Ly6G^-$ $F4/80^+$), monocytes ($CD11b^+$ $Ly6G^-$ $CD115^+$), and eosinophils ($CD11b^+$ $Ly6G^-$ $F4/80^-$ $CD115^-$ $SiglecF^+$) were shown.
Error bars represent mean ± SEM. N = 6. Absolute cell number per animal was plotted. Statistical significance was determined by unpaired two-tailed Student's *t* test. Ns: non-significant.

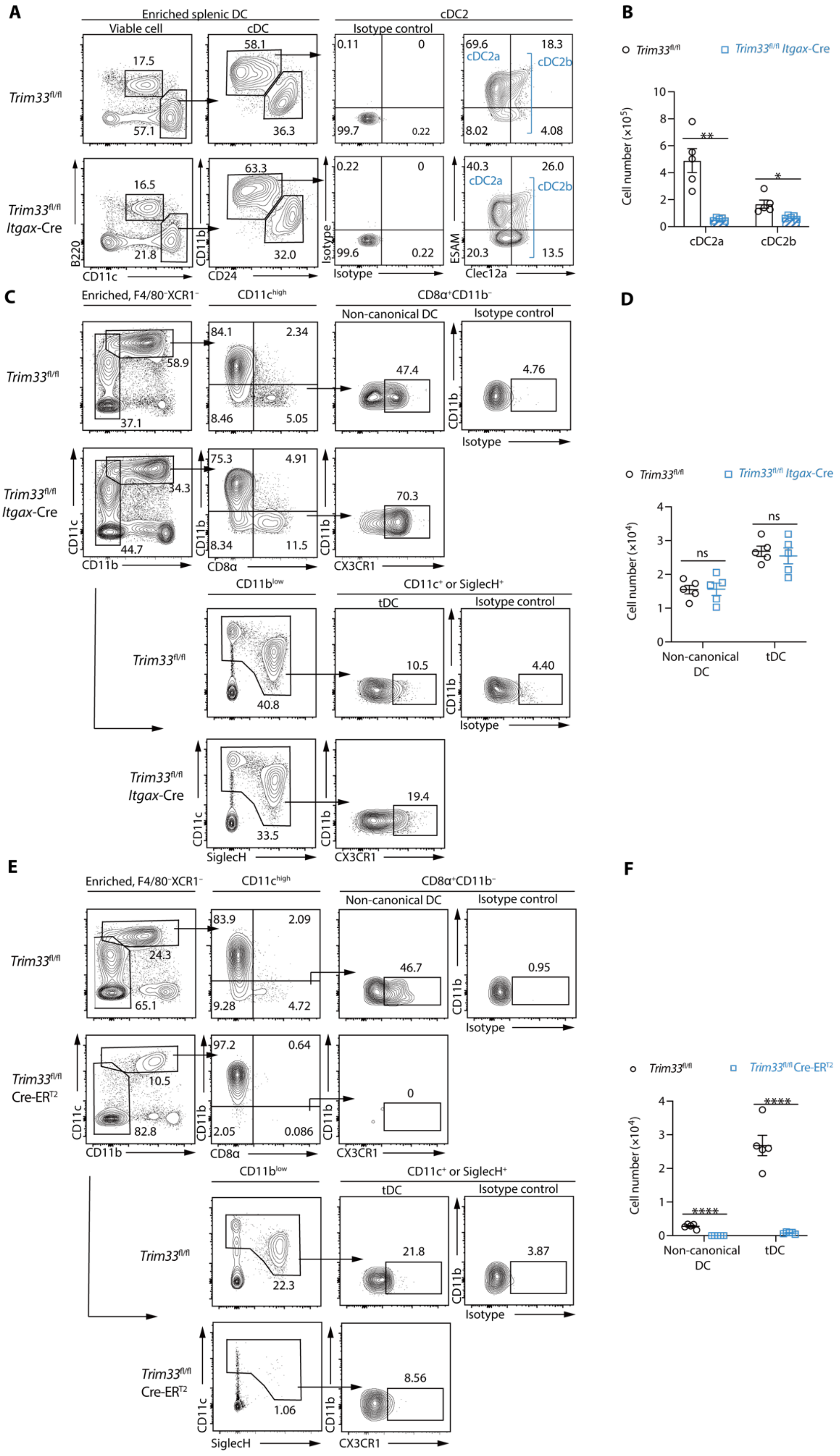

Fig. S6. Analysis of newly identified DC subsets in indicated genotypes.

(A-B) Flow cytometric analysis of Clec12a$^-$ESAM$^+$ cDC2a and Clec12a$^+$ cDC2b in *Trim33*$^{fl/fl}$ and *Trim33*$^{fl/fl}$ *Itgax*-Cre mice. Cells were pre-enriched for DC. N = 5.
(C-D) Flow cytometric analysis of non-canonical DC and tDC in *Trim33*$^{fl/fl}$ and *Trim33*$^{fl/fl}$ *Itgax*-Cre mice. Cells were pre-enriched for DC. N = 5.
(E-F) Flow cytometric analysis of non-canonical DC and tDC in Tamoxifen-treated *Trim33*$^{fl/fl}$ and *Trim33*$^{fl/fl}$ Cre-ER^{T2} mice. Cells were pre-enriched for DC. N = 5.
Error bars represent mean ± SEM. Absolute cell number per animal was plotted. Statistical significance was determined by unpaired two-tailed Student's *t* test. Ns: non-significant, * $P < 0.05$, ** $P < 0.01$, **** $P < 0.0001$.

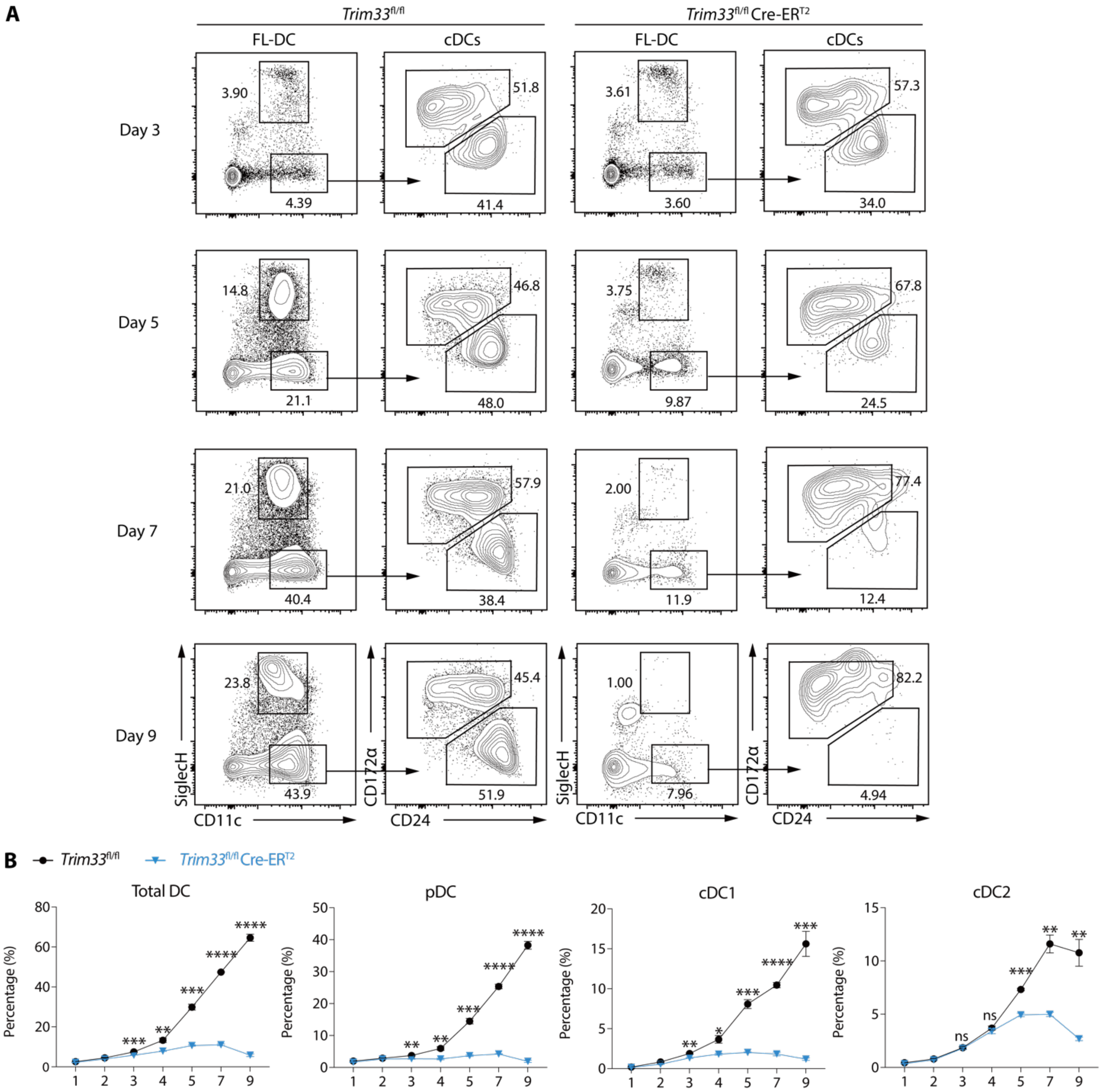

Fig. S7. Flow cytometric analysis of DC subsets generated in Flt3L cultures at different time points.
(A) Representative flow cytometric analysis of DC subsets generated from BM cells in cultures at indicated time points. 1.5 × 10^5 BM cells of indicated genotypes were cultured with 200 ng/mL Flt3L and 1 μM 4-OHT. DC generation was tested on indicated days of culture. Numbers indicate percentages of parent. Pre-gate: viable cells. pDC: $SiglecH^+$ $CD11c^{int}$, cDC: $SiglecH^-$ $CD11c^+$, cDC1: $SiglecH^-$ $CD11c^+$ $CD24^+$ $CD172\alpha^-$, cDC2: $SiglecH^-$ $CD11c^+$ $CD24^-$ $CD172\alpha^+$.
(B) Percentages of different DC subsets generated from *in vitro* Flt3L cultures at indicated timepoints. N = 3. Bars represent mean ± SEM. Representative data of 3 independent experiments are shown. Data were analyzed using two-tailed Student's t-test. *P<0.05, **P<0.01, ***P<0.001, ****P<0.0001.

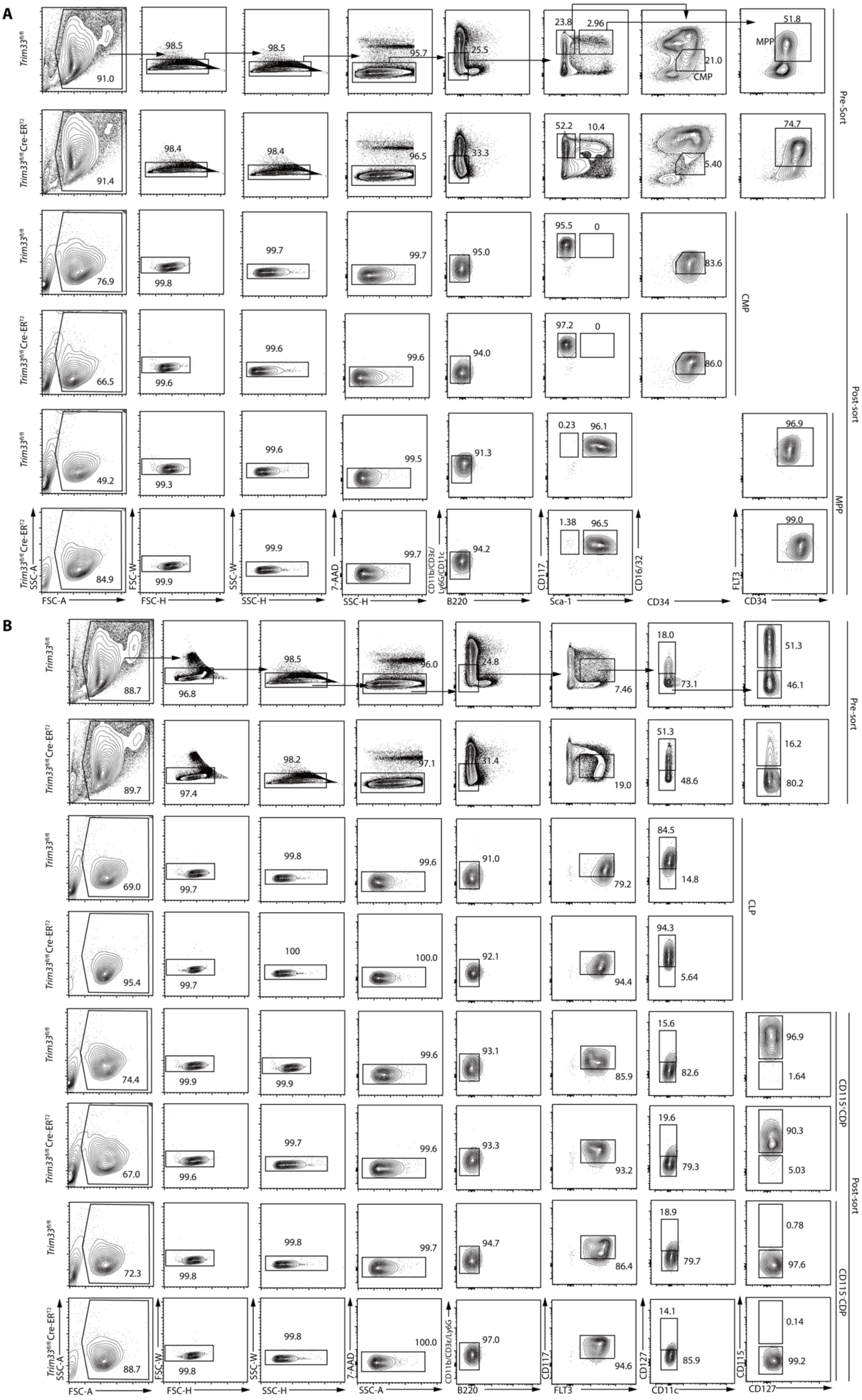

Fig. S8. Post-sort validation of purified DC progenitors.

(A) Representative validation of FACS-purified MPP and CMP of indicated genotypes. MPPs: $Lin^{-}Sca\text{-}1^{+}CD117^{hi}CD34^{+}FLT3^{+}$, CMPs: $Lin^{-}Sca\text{-}1^{-}CD117^{hi}CD34^{+}CD16/32^{lo}$.

(B) Representative validation of FACS-purified CLP and CDP of indicated genotypes. $CD115^{+}$CDPs: $Lin^{-}CD11c^{-}CD127^{-}CD117^{int}FLT3^{+}CD115^{+}$, $CD115^{-}$CDPs: $Lin^{-}CD11c^{-}CD127^{-}CD117^{int}FLT3^{+}CD115^{-}$, CLPs: $Lin^{-}CD11c^{-}CD117^{+}$ $FLT3^{+}CD127^{+}$. Numbers adjacent to gates indicated percentages of parent population.

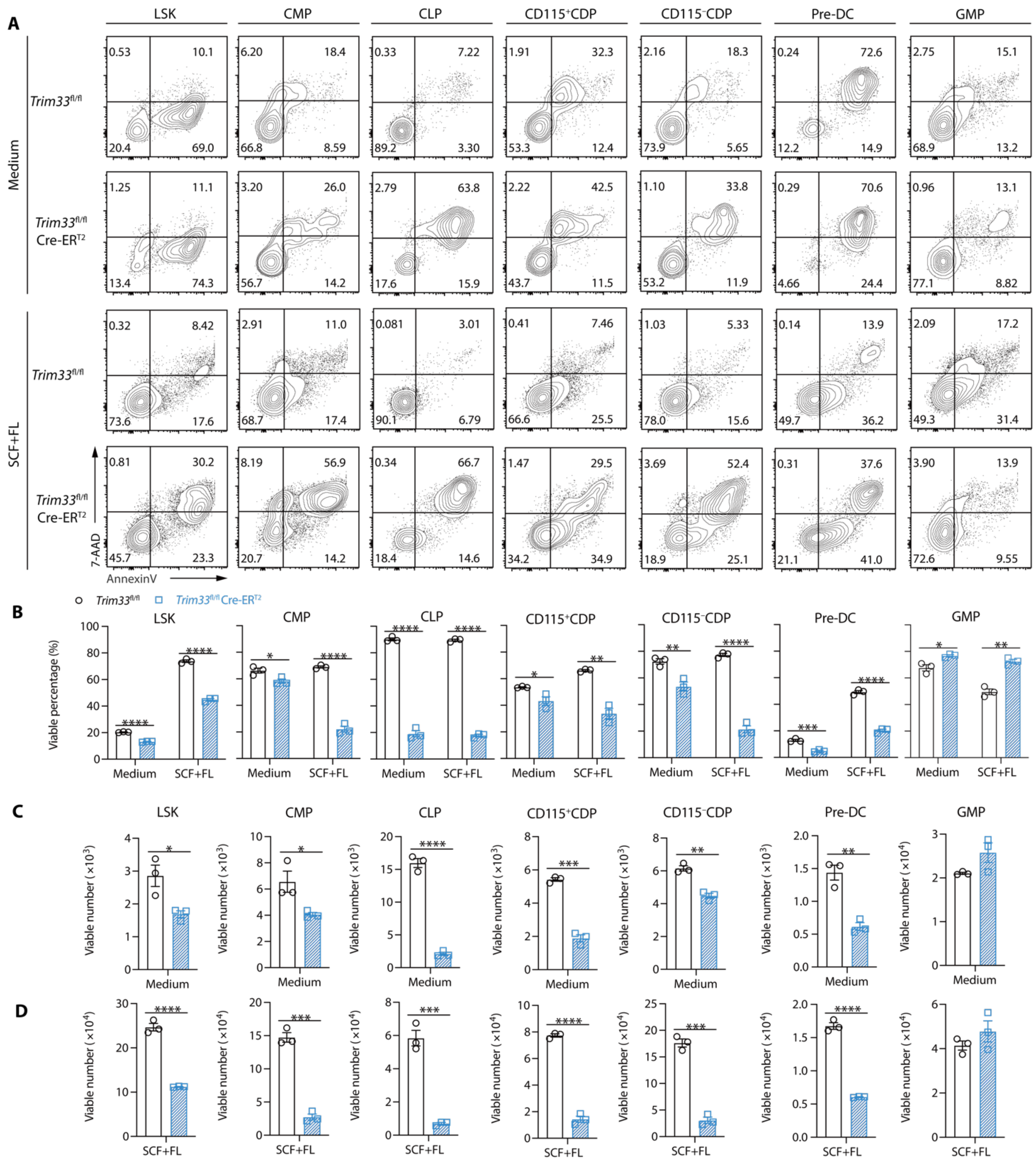

Fig. S9. Survival analysis of TRIM33-deficient progenitors.

(A) Survival analysis of cultured progenitors of indicated genotypes by flow cytometry. LSKs, CMPs, CLPs, CD115+CDPs, CD115–CDPs, pre-DCs and GMPs were purified from *Trim33*fl/fl or *Trim33*fl/fl Cre-ERT2 mice BM by FACS. 2.5×10⁴ cells/well were cultured in 1 µM 4-OHT-containing complete RPMI 1640 medium with or without 100 ng/mL SCF and 30 ng/mL Flt3L (FL) for 5 days before flow cytometric analysis with Annexin V/7-AAD double staining.

(B-D) Percentages (B) and numbers (C, D) of viable (Annexin V– 7-AAD–) cells in indicated cultures. N = 3.

Data are representative of 2 independent experiments. Error bars represent mean ± SEM. Statistical

significance was determined by unpaired two-tailed Student's *t* test. ns: $P > 0.05$. * $P < 0.05$, ** $P < 0.01$, *** $P < 0.001$, **** $P < 0.0001$.

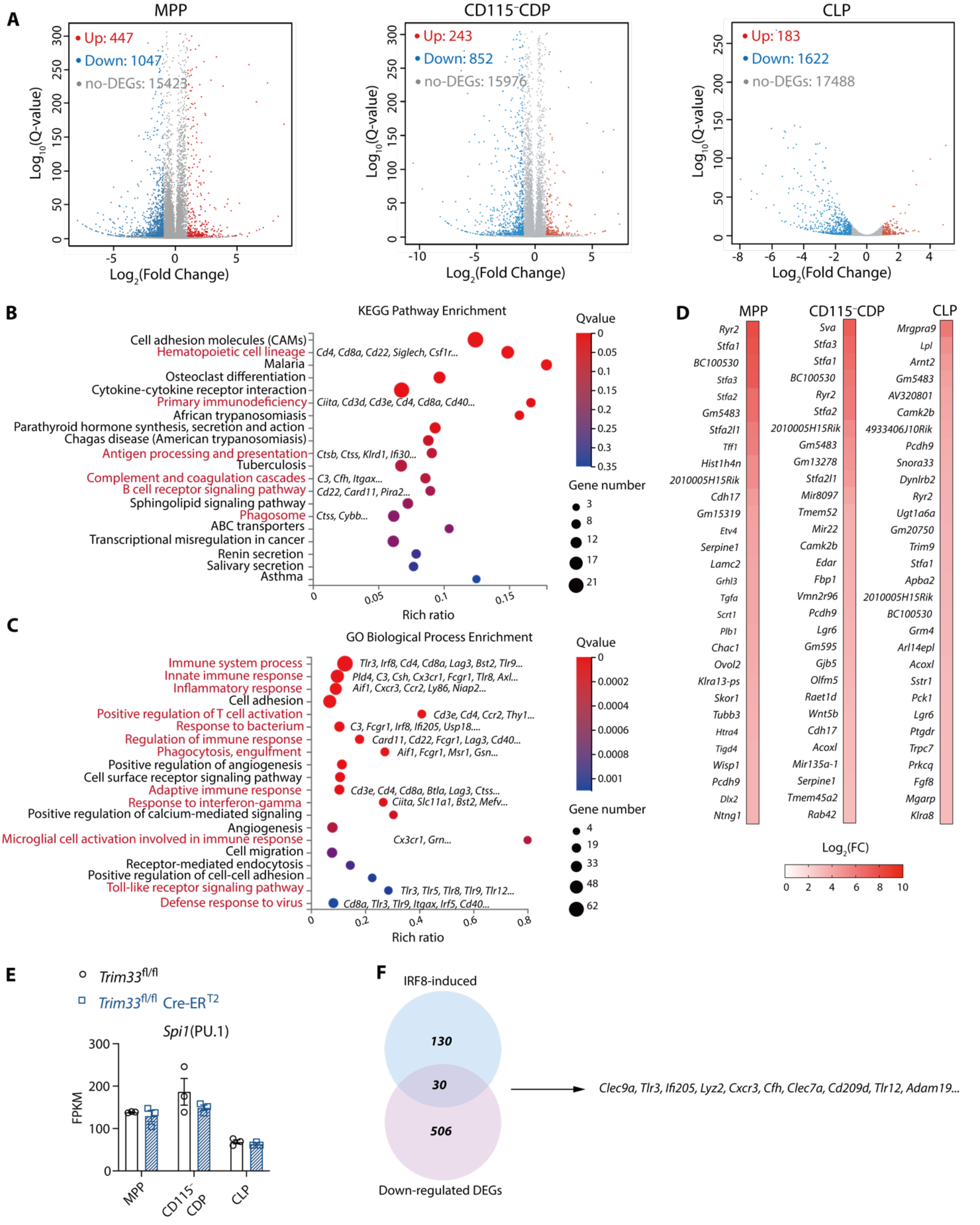

Fig. S10. RNA-seq analysis detected DEGs in WT and TRIM33-deficient DC progenitors.

(A) Volcano plots indicate DEGs in *Trim33*$^{fl/fl}$ (WT) and *Trim33*$^{fl/fl}$ Cre-ER^{T2} (KO) MPP, CD115^{-}CDP and CLPs. Analyses for differentially expressed genes were performed with DESeq for MPP and CD115^{-}CDP with threshold of Log_2 |Fold Change| ≥ 1, Q-value ≤ 0.001. DESeq2 was used for CLP with a threshold of

Log_2 |Fold Change| ≥ 1, Q-value ≤ 0.05.
(B) KEGG pathway enrichment of the DEGs shared among the progenitors. Exemplifying genes in the pathways were listed.
(C) Gene ontogeny (GO) biological process enrichment of the DEGs shared among the progenitors. Exemplifying genes in the pathways were listed.
Pathways and processes associated with hematopoiesis and immune responses were labeled in red.
(D) Top up-regulated genes in indicated KO progenitors comparing to WT. Fold change (FC) of FPKM was plotted.
(E) Expression of *Spi1* in WT and KO progenitors detected by RNA-seq. FPKM was plotted.
(F) Venn graph indicating overlap between IRF8-induced genes and the down-regulated DEGs.

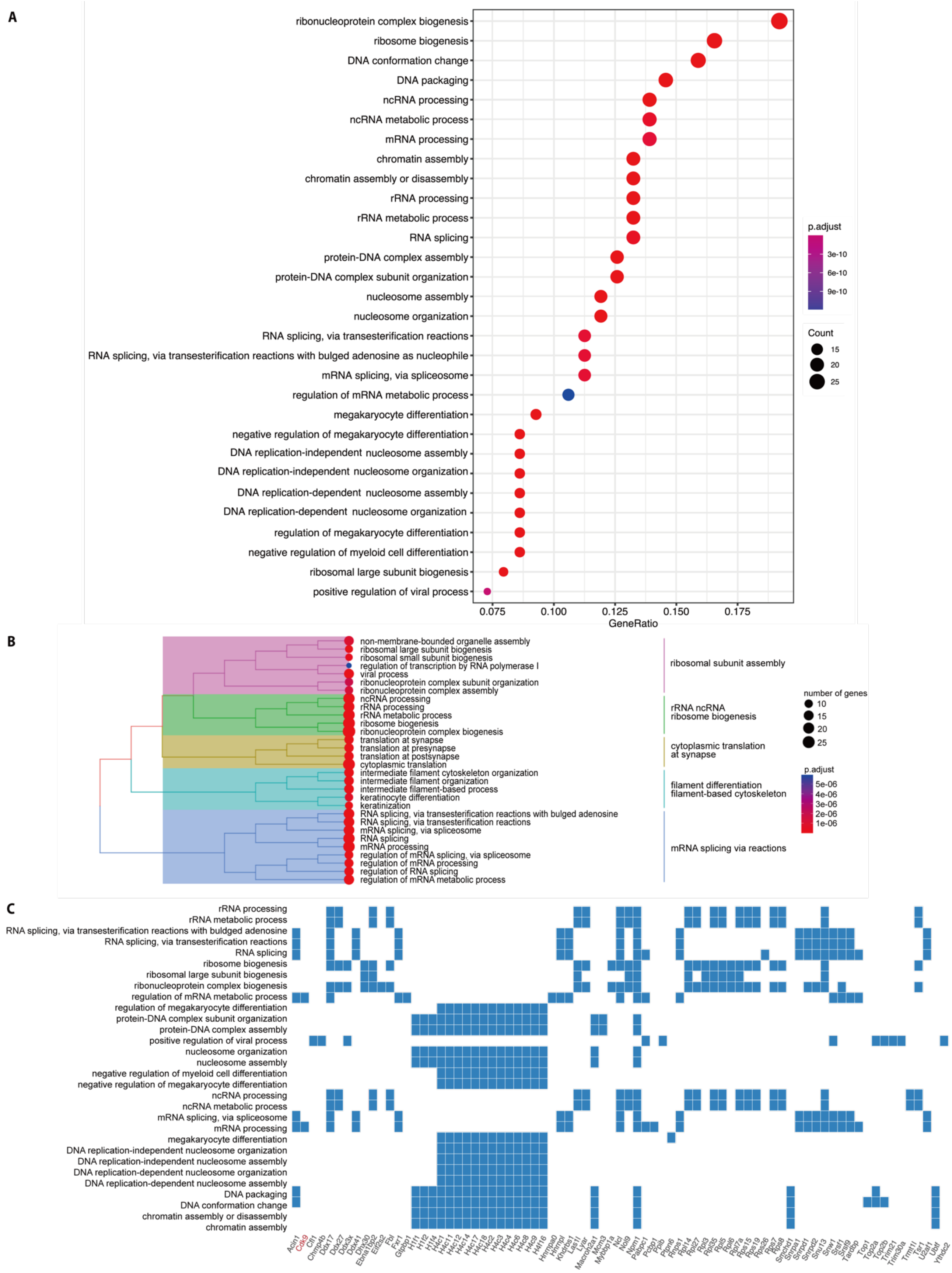

Fig. S11. Analysis of TRIM33-interacting proteins detected by co-IP/MS.

(A) Gene ontogeny (GO) biological process enrichment of the TRIM33-interacting proteins. Top 30 terms were shown.

(B) Treeplot clustering of enriched GO terms.

(C) Heatmap of enriched GO terms. A colored block indicates involvement of a specific gene in a corresponding term.

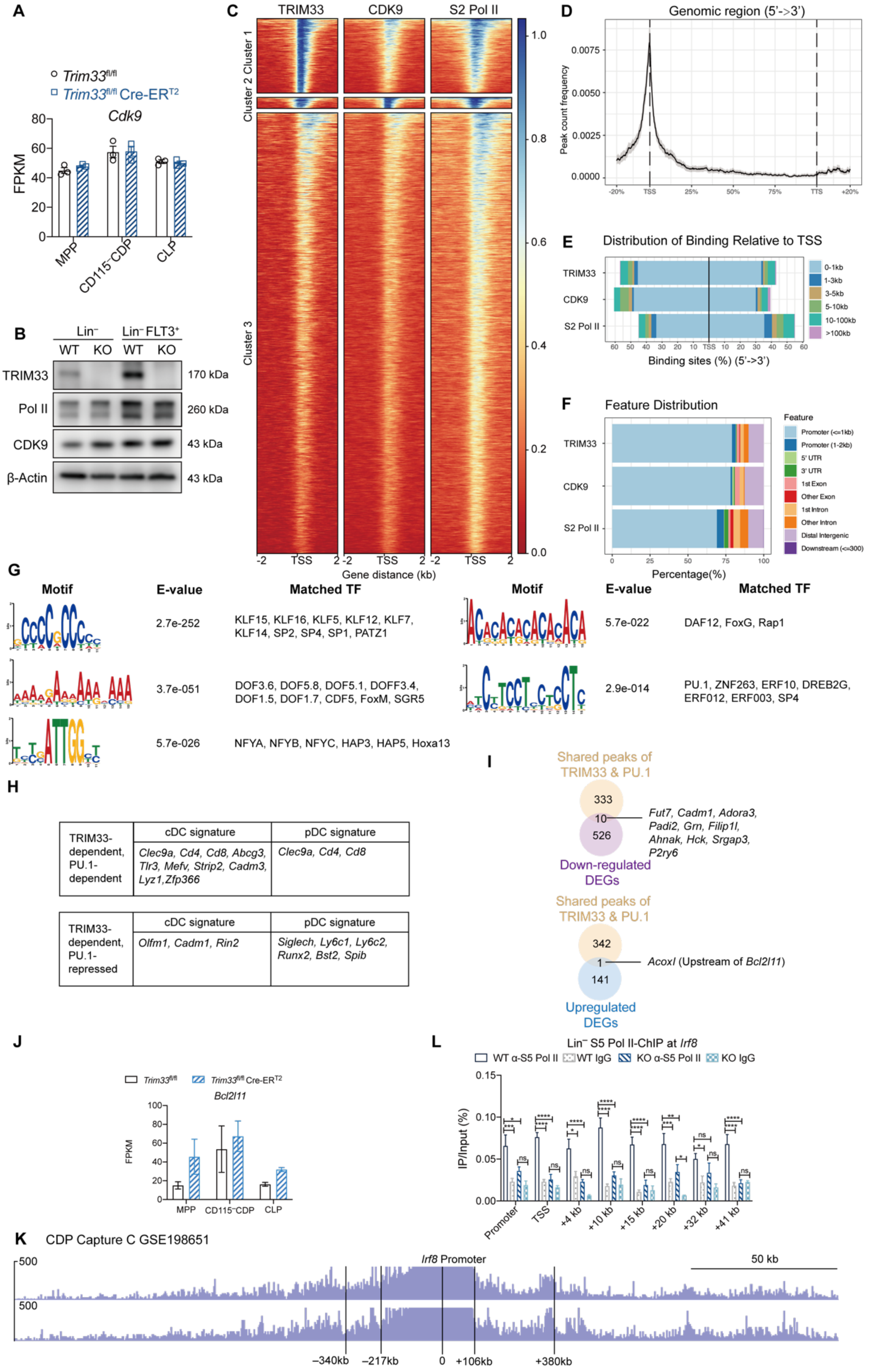

Fig S12. Additional profile of TRIM33 genome-wide binding sites in CDP.

(A) RNA-seq analysis detected FPKM of *Cdk9* in indicated BM progenitors of Tamoxifen-treated *Trim33*$^{fl/fl}$ and *Trim33*$^{fl/fl}$ Cre-ER^{T2} mice. N = 3.
(B) Western blot analysis of CDK9, Pol II, and TRIM33 levels in Tamoxifen-treated *Trim33*$^{fl/fl}$ (WT) and *Trim33*$^{fl/fl}$ Cre-ER^{T2} (KO) BM Lin^{-} and Lin^{-} FLT3^{+} cells.
(C) Heatmap representation of TRIM33, CDK9, and S2 Pol II CUT-Tag peaks around transcription start site (TSS) in CDP.
(D) Count frequency of TRIM33 CUT-Tag peaks at gene body regions. Gray shades indicate 95% confidence interval.
(E) Distribution of TRIM33, CDK9, and S2 Pol II binding sites disctances to TSS.
(F) Genomic feature distributions of TRIM33, CDK9, and S2 Pol II in CDP.
(G) Motif enrichment analysis of TRIM33 CUT&Tag peaks in CDP performed by MEME-ChIP.
(H) DC signature genes among those under dual transcription regulation of TRIM33 and PU.1 progenitors determined by Fig. 5E.
(I) Intersection between genes annotated from TRIM33 and PU.1 shared peaks in CDP and RNA-seq-detected DEGs in progenitors.
(J) The levels of *Bcl2l11* mRNA in indicated progenitors detected by RNA-seq. FPKM was shown. N = 3.
(K) Tracks of Capture C tracks in CDP using *Irf8* promoter as viewpoint. Distances to *Irf8* promoter was labeled. Data were acquired from GSE198651.
(L) ChIP-qPCR analysis of S5 Pol II occupancy at different regions of *Irf8* gene in BM Lin^{-} cells of Tamoxifen-treated *Trim33*$^{fl/fl}$ (WT) and *Trim33*$^{fl/fl}$ Cre-ER^{T2} (KO) mice. N = 3-4. Data are representative of 2 independent experiments.
Bars represent mean ± SEM. Data were analyzed using two-way ANOVA followed by Bonferroni's multiple comparison test (L). **P*<0.05, ***P*<0.01, ****P*<0.001, *****P*<0.0001.

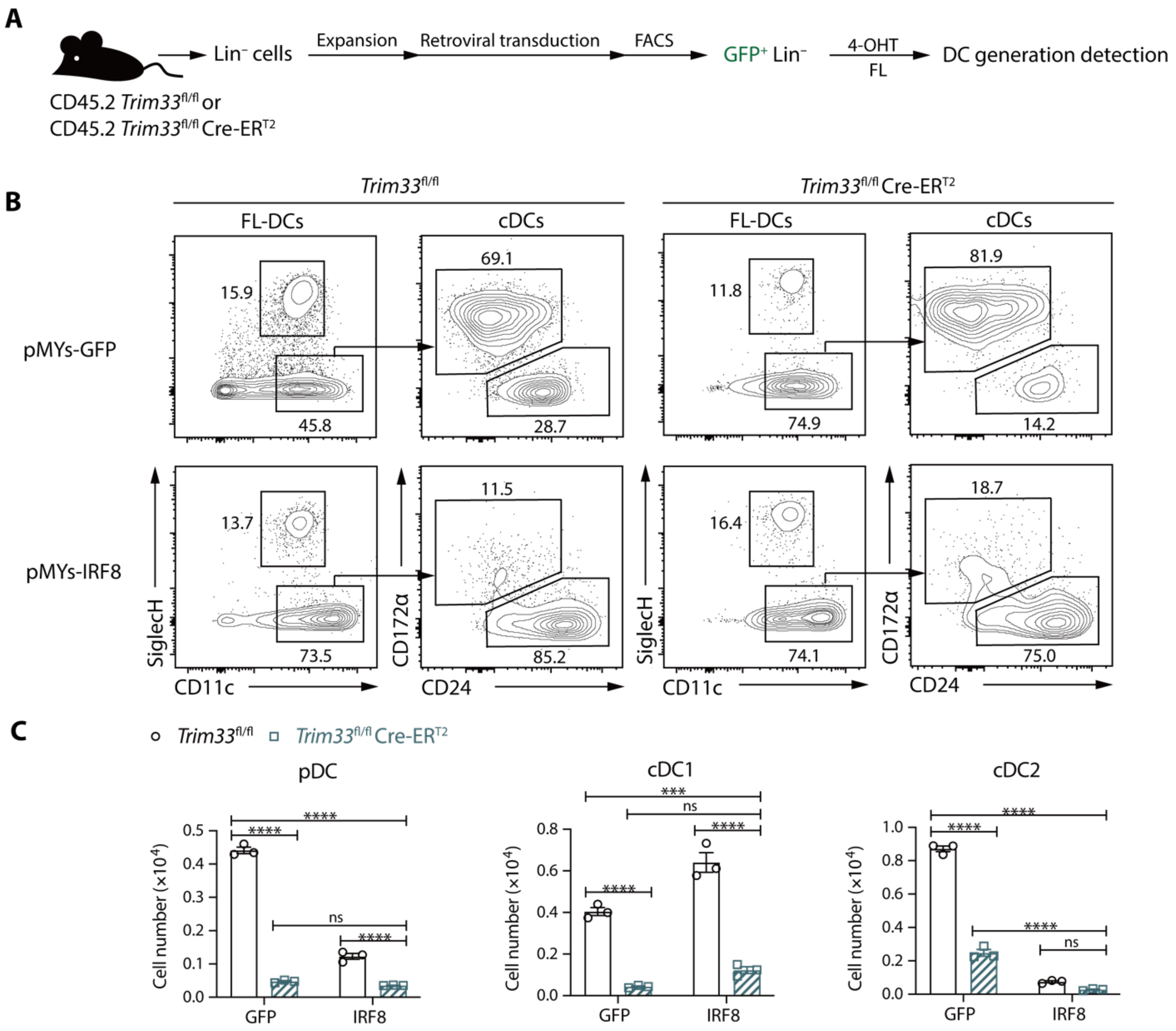

Fig. S13. DC generation from *Irf8*-overexpressing TRIM33-deficient BM Lin⁻ cells in Flt3L cultures. **(A)** Schematic representation for *in vitro* DC generation from retrovirally transduced Lin⁻ cells. Lin⁻ precursors were sorted from *Trim33*fl/fl and *Trim33*fl/fl Cre-ERT2 BM, expanded with 200 ng/mL Flt3L overnight, and retrovirally transduced with control pMYs-GFP or pMYs-IRF8 constructs. CD45.2 GFP⁺ cells were FACS-purified 48 h post transduction, seeded with CD45.1 feeder cells, cultured in media supplemented by 200 ng/mL Flt3L and 1 μM 4-OHT for 5 additional days, and detected for DC generation. **(B and C)** Flow cytometry analysis of DCs generated from *Irf8*-overexpressing Lin⁻ cells of indicated genotypes. Cells were pre-gated on viable, CD45.2⁺ GFP⁺. Numbers adjacent to gates indicate percentages of parent. Numbers adjacent to gates indicate percentages of parent. Plots in (C) indicate numbers of DCs generated from per 1×10^4 input Lin⁻ cells. N = 3.

Bars represent mean ± SEM. Representative data of 2 independent experiments are shown. Data were analyzed using two-way ANOVA followed by Bonferroni's multiple comparison test. Ns: non-significant, ****P*<0.001, *****P*<0.0001.

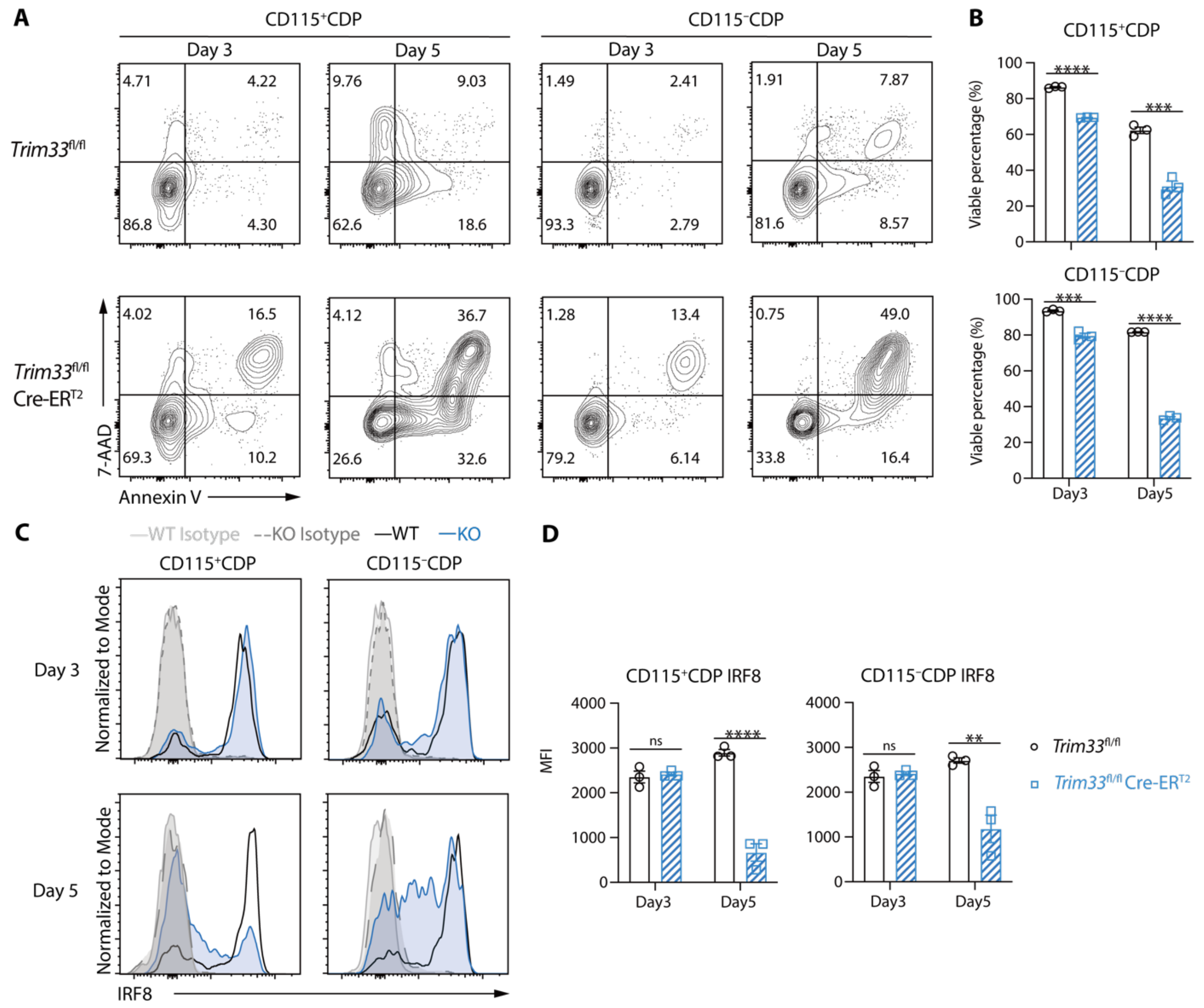

Fig. S14. Time course analysis of CDP cultures *in vitro*.

(A and B) Cell viability analysis of CDP cultures at indicated time points. CDPs were sorted from *Trim33*$^{fl/fl}$ and *Trim33*$^{fl/fl}$ Cre-ER^{T2} BM. 2.5×10^{4} cells/well were cultured with 100 ng/mL SCF and 30 ng/ml Flt3L for 3-5 days before subjected to Annexin V/7-AAD or intracellular IRF8 staining.

(C and D) Mean fluorescent intensity (MFI) of IRF8 in CDP cultures at indicated time points.

Bars represent mean ± SEM. Representative data of 2 independent experiments are shown. N = 3. Data were analyzed using unpaired two-tailed Student's *t* test. ns: $P > 0.05$. ** $P < 0.01$, *** $P < 0.001$, **** $P < 0.0001$.

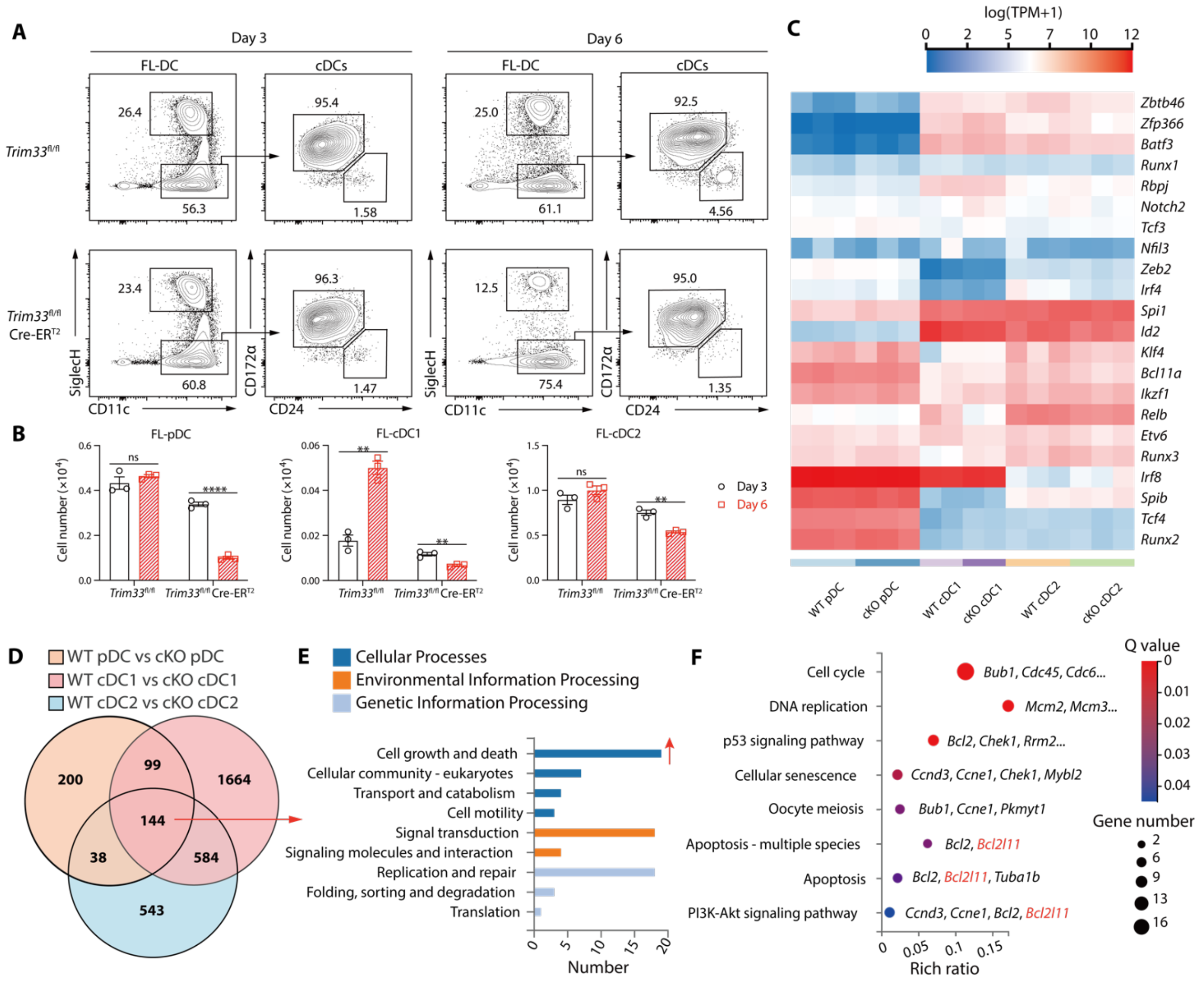

Fig. S15. Transcriptome analysis of TRIM33-deficient terminally differentiated DCs with defective maintenance.

(A and B) Flow cytometry analysis of FL-DCs generated from pre-DCs in Flt3L cultures. 5×10^4 pre-DCs isolated from the BM of *Trim33*$^{fl/fl}$ (WT) or *Trim33*$^{fl/fl}$ Cre-ER^{T2} (KO) mice were seeded with 1.5×10^5 CD45.1 BM feeders per well in 96-well plates and cultured in complete RPMI 1640 media supplemented with 1 μM 4-OHT and 200 ng/mL Flt3L for 3-6 days. Viable, CD45.2$^+$ cells were pre-gated. Numbers adjacent to gates represent percentages of parent population. Numbers of DCs generated from per 1×10^4 input pre-DCs are plotted in (D). N = 3. Bars represent mean ± SEM. Representative data of 2 independent experiments are shown. Data were analyzed using unpaired two-tailed Student's *t* test. ns: $P > 0.05$, ** $P < 0.01$.

(C) RNA-Seq analysis of *Trim33*$^{fl/fl}$ (WT) and *Trim33*$^{fl/fl}$ *Itgax*-Cre (cKO) splenic DCs. Heatmap showing the expression of DC differentiation-associated genes. TPM (transcripts per kilobase of exon model per million mapped reads) for each gene was shown. Each column represents a biological repeat, which was obtained from 4-5 WT mice or 10-11 cKO mice.

(D) Venn diagrams of RNA-Seq-detected differentially expressed genes (DEGs, Q < 0.01, $|\log_2 FC| > 0.5$) by WT and cKO splenic DCs.

(E) KEGG pathway classification of the common DEGs among pDCs, cDC1s and cDC2. Arrow indicates upregulation of genes involved in the pathway.

(F) KEGG pathway enrichment of the common DEGs in the cell growth and death secondary classification. Exemplifying genes of each pathway are listed.

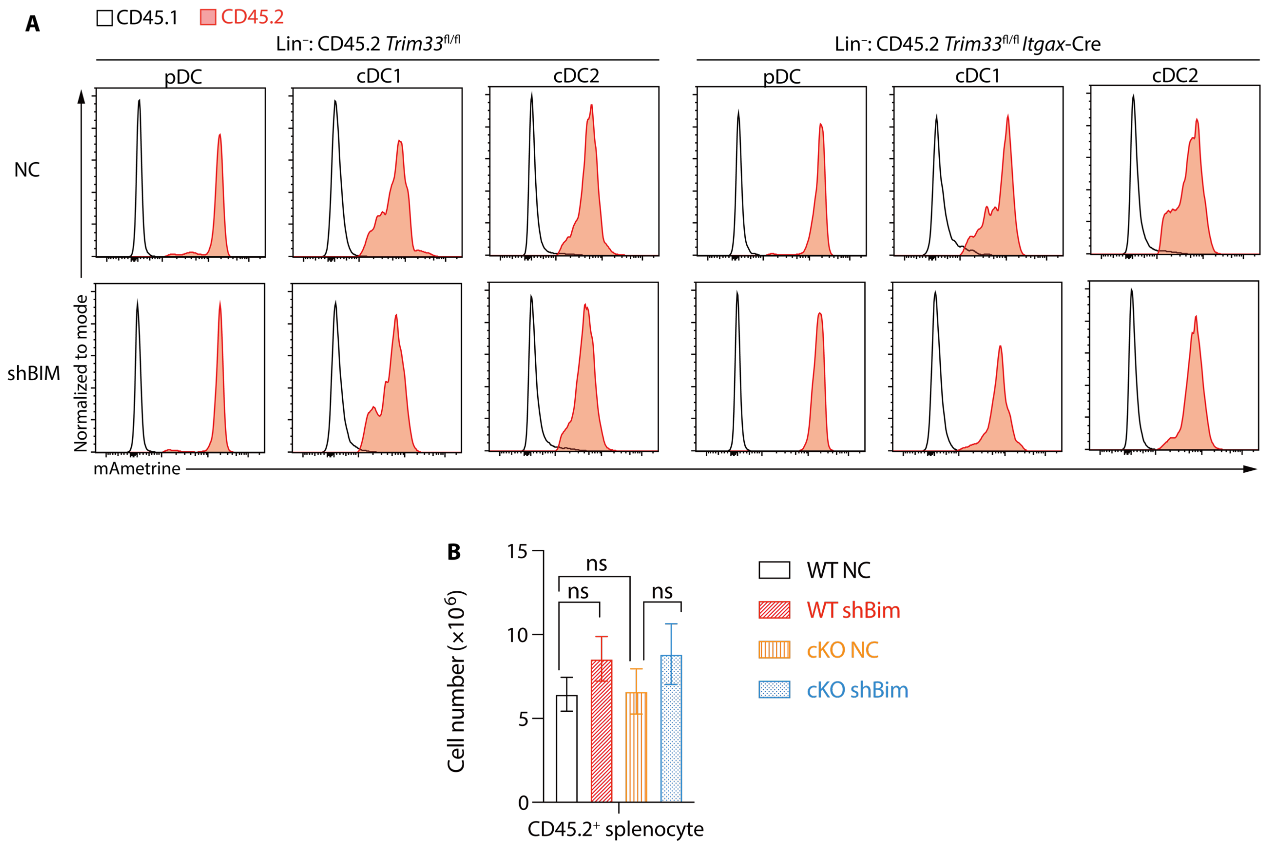

Fig. S16. Additional properties of CD45.2^{+} cells reconstituted from BM Lin^{-} cells *in vivo*.

(a) Flow cytometry plots indicating retained vector expression by *in vivo* differentiated CD45.2^{+} DCs 4 weeks post reconstitution. Plots are representative of 3 independent experiments.

(b) Numbers of reconstituted CD45.2^{+} mAmetrine^{+} splenocytes. N = 6-10. Error bars represent mean ± SEM. Data were analyzed by multiple *t* tests. ns: non-significant.

Table S7. Yield of DC progenitor or precursor per mouse by FACS.

Cell type	*Trim33*$^{fl/fl}$ or untreated *Trim33*$^{fl/fl}$ Cre-ER^{T2}	Tamoxifen-treated *Trim33*$^{fl/fl}$ Cre-ER^{T2}
MPP	(2-3)×10^4	(6-10)×10^4
CMP	(1-1.5)×10^5	(0.3-0.5)×10^5
CD115$^+$CDP	(1.5-2)×10^4	(0.3-1)×10^4
CD115$^-$CDP	(3-5)×10^4	(10-15)×10^4
CLP	(1.5-3)×10^4	(5-15)×10^4
Pre-DC	(8-10)×10^4	(1-2)×10^4
