# Supplementary material for: TRIM33 plays a critical role in regulating dendritic cell differentiation and homeostasis by modulating Irf8 and Bcl2l11 transcription
Source: Cell Mol Immunol. 2024 May 31;21(7):752–69. doi: 10.1038/s41423-024-01179-1 (PMC11214632; doi:10.1038/s41423-024-01179-1)

Figure 4F

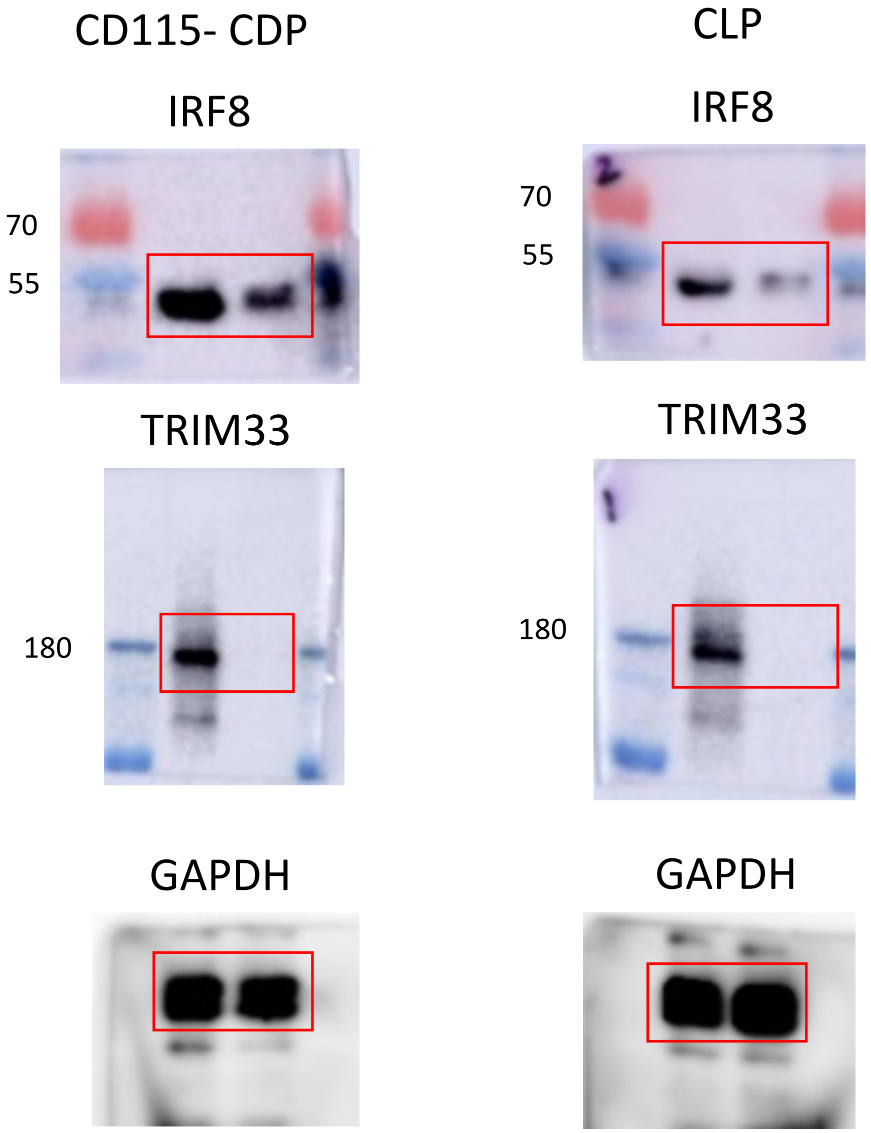

CD115- CDP
CLP
IRF8
70
55
IRF8
70
55
TRIM33
180
TRIM33
180
GAPDH
GAPDH

Figure 5A

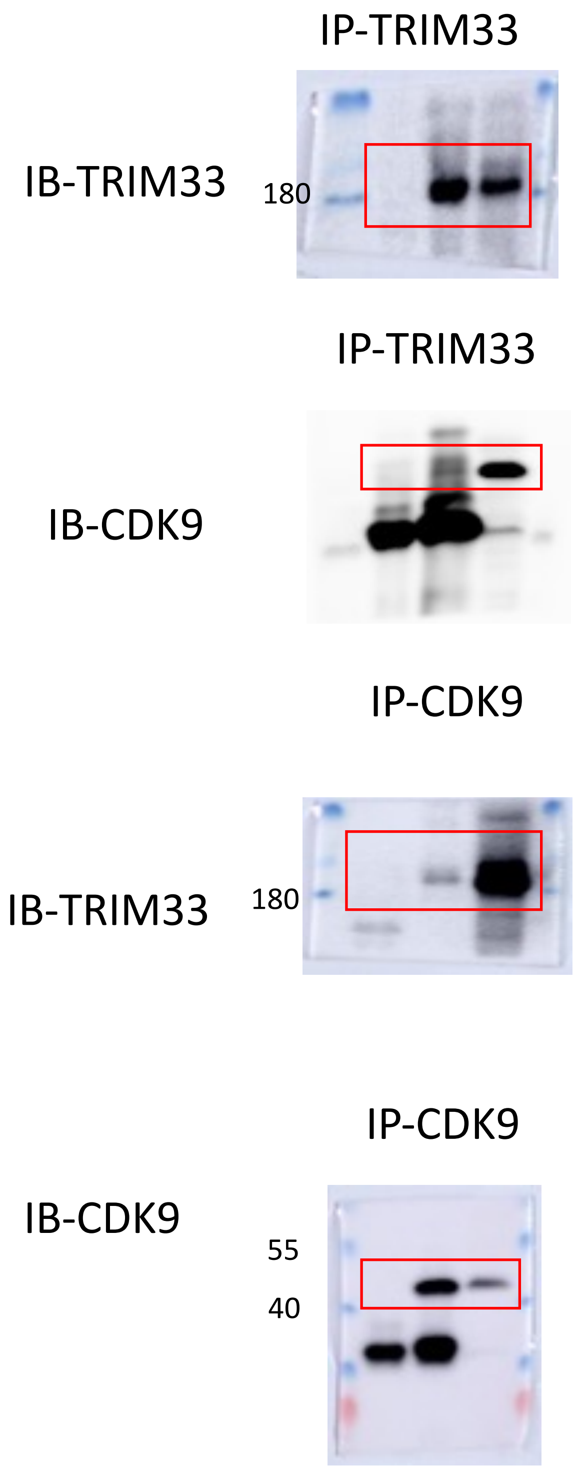

IP-TRIM33
IB-TRIM33
180
IP-TRIM33
IB-CDK9
IP-CDK9
IB-TRIM33
180
IP-CDK9
IB-CDK9
55
40

Figure 6G

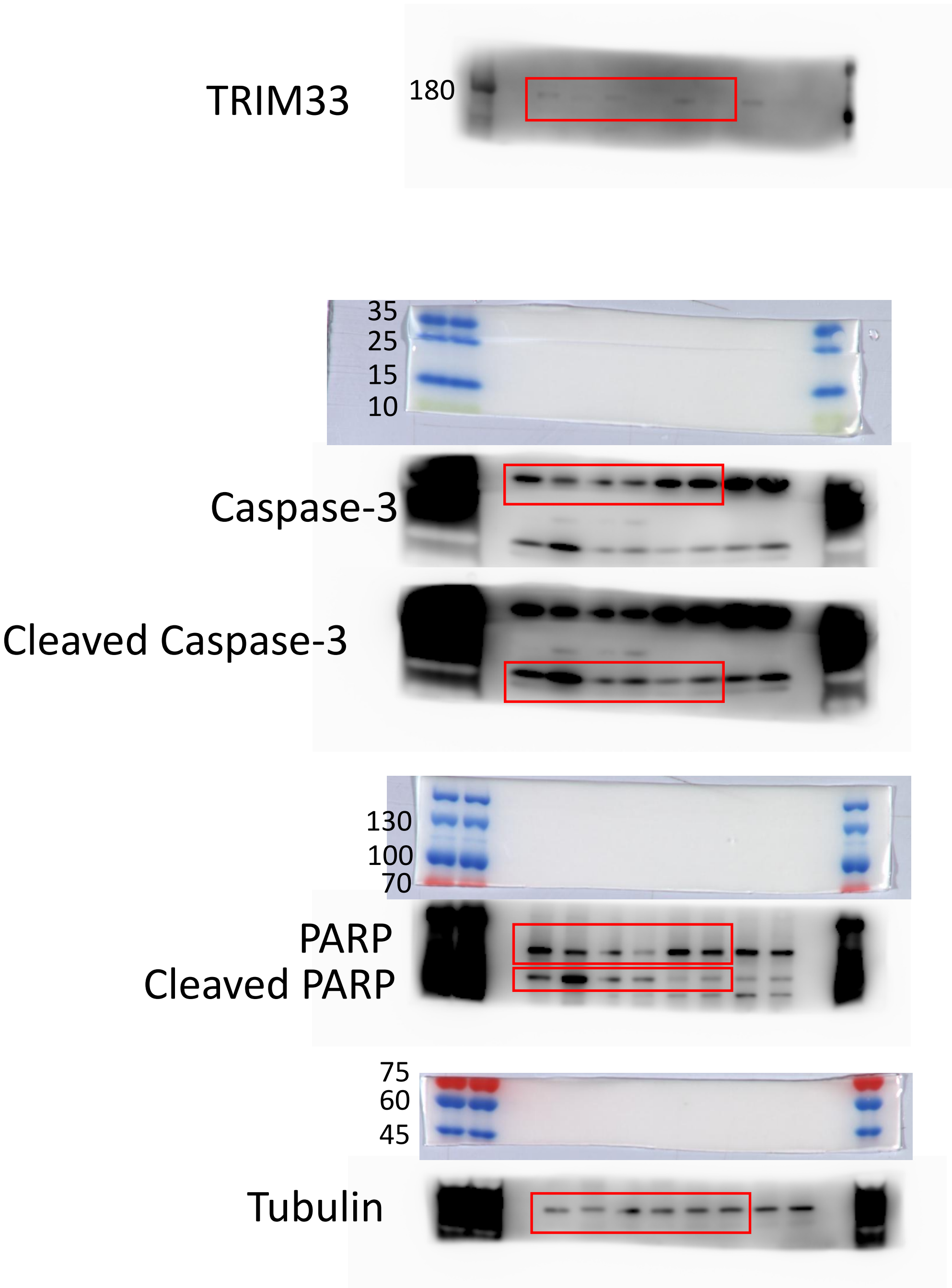

TRIM33
180
35
25
15
10
Caspase-3
Cleaved Caspase-3
130
100
70
PARP
Cleaved PARP
75
60
45
Tubulin

Figure 7E

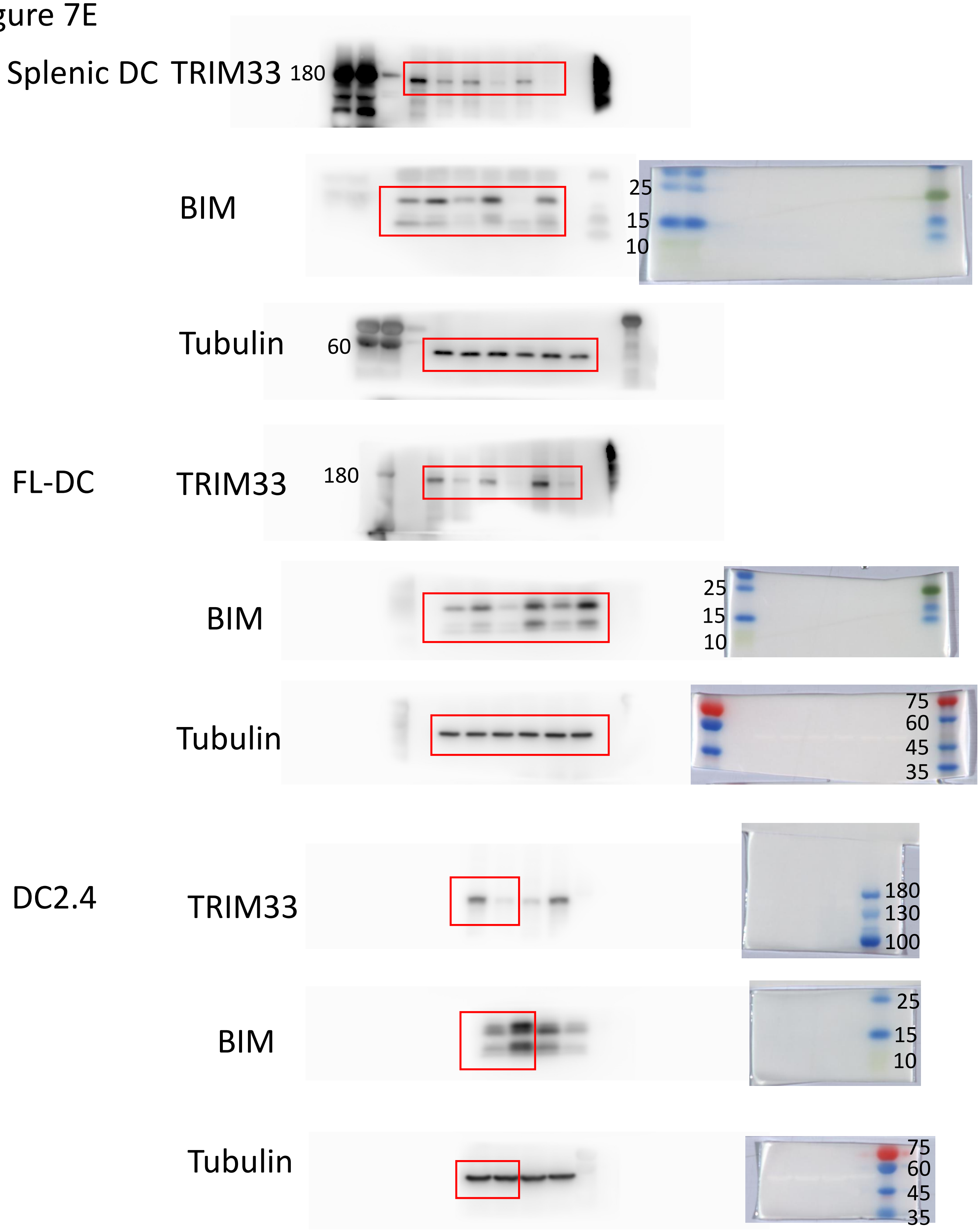

Splenic DC
TRIM33
180
BIM
25
15
10
Tubulin
60
FL-DC
TRIM33
180
BIM
25
15
10
Tubulin
75
60
45
35
DC2.4
TRIM33
180
130
100
BIM
25
15
10
Tubulin
75
60
45
35

Figure 7I

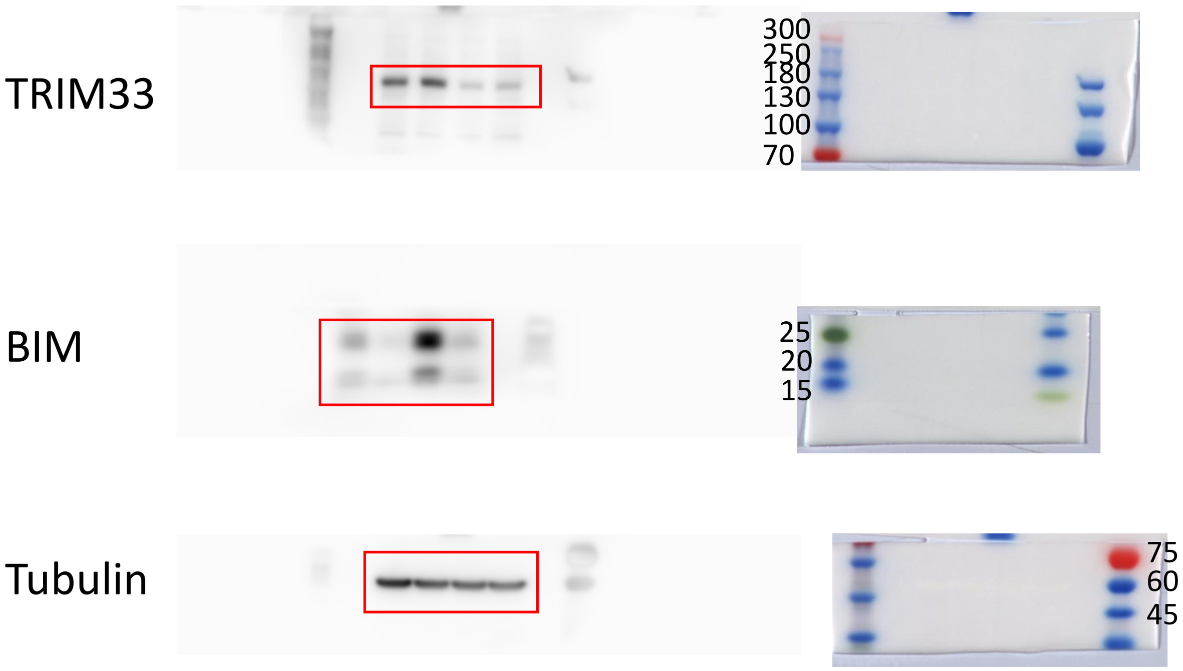

Figure 8B

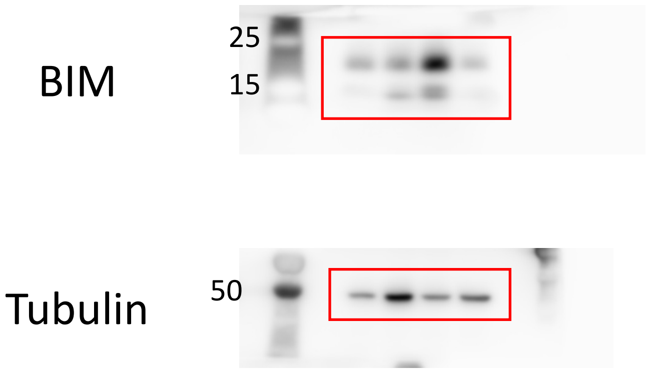

Figure 8F

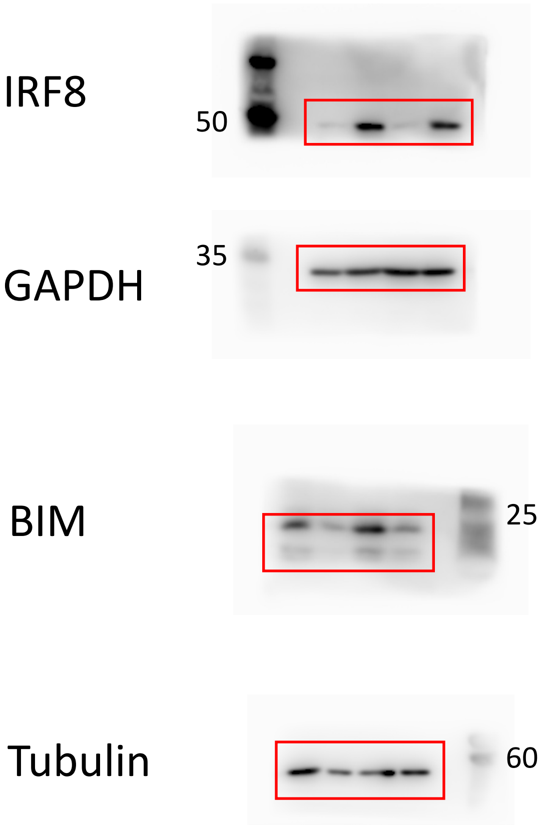

Figure S1E

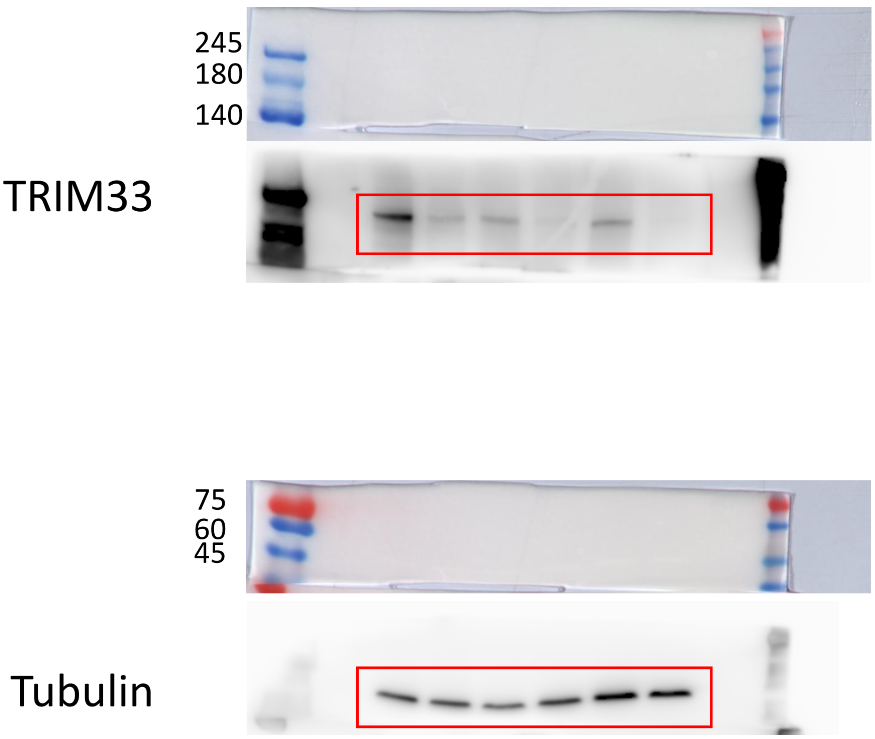

Figure S12B

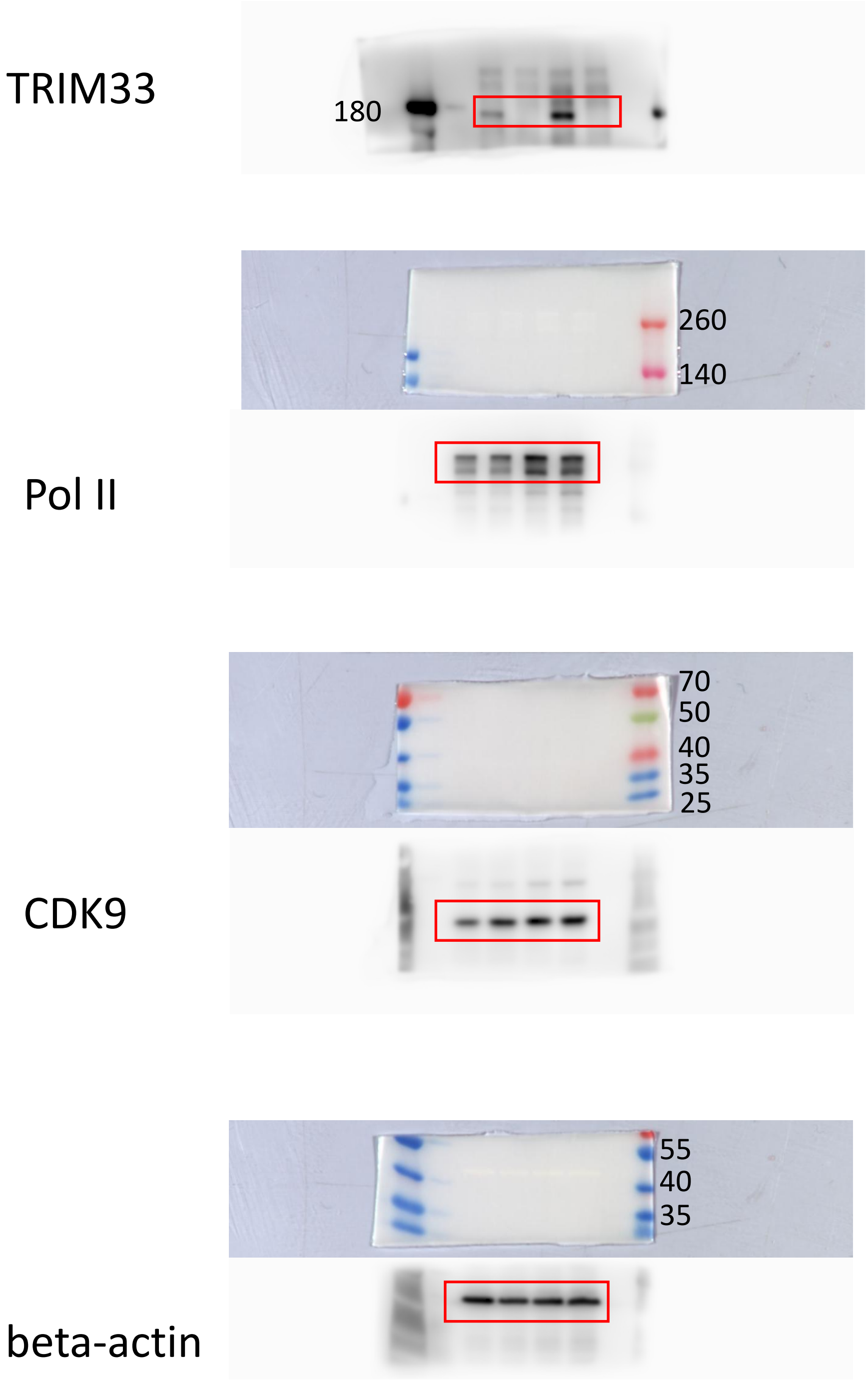

Supplement: Supplementary file 9 — Uncropped Western blot gels [file 41423_2024_1179_MOESM9_ESM.pdf]
